# Personalized care of paediatric drug‐resistant epilepsy in Africa: A single‐centre pilot study utilizing mobile health and genetic testing

**DOI:** 10.1111/dmcn.16478

**Published:** 2025-08-20

**Authors:** Ian S. Olivier, Karen Fieggen, Sandra Komarzynski, Elin H. Davies, Irene Muchada, Caitlin McIntosh, Alina Esterhuizen, Richard J. Burman, Jo M. Wilmshurst, and the University of Cape Town (UCT) Human Genetics Precision Medicine Epilepsy Group, Nicholas Loxton, Shrinav R Dawlatt, Akshay Vanmali

**Affiliations:** ^1^ Neuroscience Institute University of Cape Town Cape Town South Africa; ^2^ Department of Psychiatry and Mental Health University of Cape Town, Observatory South Africa; ^3^ Division of Human Genetics, Department of Medicine University of Cape Town, Observatory South Africa; ^4^ Aparito Ltd Wrexham UK; ^5^ National Health Laboratory Service Groote Schuur Hospital Cape Town South Africa; ^6^ Department of Paediatric Neurology Red Cross War Memorial Children's Hospital Cape Town South Africa

## Abstract

**Aim:**

To evaluate personalized care or precision medicine initiatives, including mobile health (mHealth) technology and genetic screening, in a South African paediatric epilepsy clinic.

**Method:**

This exploratory prospective observational pilot study included 39 children aged 4 years or older with drug‐resistant epilepsy (ongoing seizures despite at least two antiseizure medications at adequate doses). Participants were recruited from the epilepsy service at the Red Cross War Memorial Children's Hospital in Cape Town, the largest paediatric hospital in sub‐Saharan Africa. mHealth technology – a wearable device and mobile application – allowed recording of seizures, medication, sleep, mobility, quality of life, and health visits. Genetic testing included a customized gene panel and pharmacogenomic arrays.

**Results:**

Seizure frequency, but not duration, was significantly greater in clinical records before and during the study period (8.0 and 5.5 median seizures per month respectively) compared with mHealth records of 2.0 median seizures per month (*n* = 28, *p* < 0.001, *r* = 0.64, 95% confidence interval [CI] 2.62–14.25, Wilcoxon signed‐rank test, and *n* = 21, *p* < 0.001, *r* = 0.76, 95% CI 2.25–15.75, Wilcoxon signed‐rank test respectively). Wearable devices detected decreased activity and sleep in patients compared with age‐matched typically developing peers (both *p* < 0.001, Mann–Whitney *U* test). Structural abnormalities were the most common aetiology. Pathogenic variants occurred in two different probands in *SCN1A*, one likely pathogenic variant in *GRIN2A*, and two variants of unknown significance in *GABRG2* and *GRIN2B*. Pharmacogenomic analyses identified variants of interest in *CYP2D6*, *EPHX1*, and *SCN1A*.

**Interpretation:**

Precision medicine for drug‐resistant epilepsy using mHealth and genetics may show use in a resource‐limited setting. This study, the first demonstration of precision medicine in an African paediatric setting, informs the development of diagnostic testing and provides novel insights into the lives and genetics of affected children.

## Graphical abstract

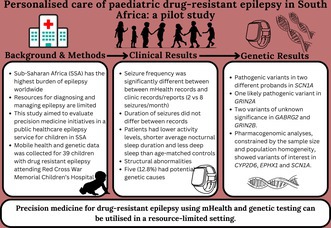

Plain language summary: https://onlinelibrary.wiley.com/doi/10.1111/dmcn.70075

**This original article is commented on by Mante on pages** 307–308 **of this issue.**

**Abbreviations**
ASM: antiseizure medication
CHU9D: Child Health Utility 9D
LMIC: lower‐middle income country
mHealth: mobile health
SSA: sub‐Saharan Africa

**What this paper adds**

- Mobile health can be used in resource‐limited settings but is limited by contextual psychosocial challenges.
- The mobile technology and clinical notes recorded different seizure frequencies.
- On average, participants were less active and slept less with lower proportions of deep sleep than age‐matched typically developing peers.
- Pathogenic variants occurred in two different probands in *SCN1A*, one likely pathogenic variant in *GRIN2A*, and two variants of unknown significance, *GABRG2* and *GRIN2B*.
- Pharmacogenomic analyses showed variants of interest in *CYP2D6*, *EPHX1*, and *SCN1A*.

Epilepsy is not only a chronic condition characterized by seizure recurrence, but also a continuing disease which often affects cognition, sensory and motor processing, mental health, and other brain functions.^1^ It affects some 50 million people worldwide, with more than 80% of these living in low‐ and middle‐income countries (LMICs).^2^, ^3^ Epilepsy ranks highest for disability‐adjusted life years in countries in sub‐Saharan Africa (SSA),^2^ and is often undiagnosed and untreated.^4^ Key risk factors in SSA include the high prevalence of central nervous system infections, perinatal insults, and head trauma.^5^, ^6^ Most cases of epilepsy present in childhood, with an incidence ranging from 41 to 187 per 100 000.^7^ The epilepsy treatment gap is high in Africa (47% in urban to 73% in rural regions), as access to diagnostic tools is limited to tertiary and private hospitals. Only 33% of treated patients are managed appropriately.^5^ Stigma, prejudice, and misconceptions also influence treatment access and adherence.^4^, ^8^

Precision medicine or personalized care aims to provide individualized, optimal, and targeted care.^9^, ^10^ Mobile or other wireless technologies (mHealth or eHealth) as well as genetic testing are widely used in health‐care management of individual patients in high‐income countries,^11^ and could also significantly contribute to a personalized approach to care in LMICs.^12^

Poor recall of seizure semiology and underestimation of seizure frequency results in suboptimal treatment.^9^, ^13^ Comorbidities, such as sleep quality and behaviour, can be challenging to document in routine clinic reviews. Our previous pilot study identified local barriers to implementing mHealth technologies in resource‐limited settings, but illustrated how mHealth could enable clinicians to observe patients outside clinic visits, and capture in real time additional aspects of health such as sleep, behaviour, and quality of life, so providing a fuller picture of the burden of disease both for the child with epilepsy and the family.^12^

Early diagnosis and appropriate treatment is important in many genetic epilepsies for targeted therapy and optimal outcomes.^14^ However, genomic approaches are still underexplored in LMICs, especially SSA.^8^, ^14^, ^15^ Individual responses to antiseizure medications (ASMs) in efficacy, optimal dosing, and adverse drug reactions are unpredictable, though largely related to the genetic aetiology.^16^, ^17^ This is particularly relevant in addressing drug‐resistant epilepsy where several mechanisms and largely unclear pharmacogenomic factors probably play a role.^16^

This pilot study aimed to evaluate the potential implementation of precision medicine initiatives via mHealth and genetic analysis in a South African public health‐care epilepsy clinic, and to describe associated novel mHealth and genetic data that can be discovered using these approaches.

## METHOD

Ethics approval was obtained from the University of Cape Town Human Research Ethics Committee (reference 767/2017). An exploratory prospective observational study design was used.

### Participants

Forty children with drug‐resistant epilepsy, defined as ongoing seizures despite at least two ASMs given at adequate doses, who were 4 years of age or older and attending the epilepsy service at the Red Cross War Memorial Children's Hospital in Cape Town were recruited. Written informed consent from parents or guardians and assent from children cognitively functioning over the age of 8 years were taken.

### mHealth technology‐based data

Patients received standard clinical care with documentation of seizures and relevant events using a paper‐based diary for a duration of 3 months. Thereafter, the mHealth platform was introduced for a duration of 6 months. An illustration of this study process and sequence can be found in Appendix S1. The mHealth initiative included a customized mobile application downloaded to the phone for the study and paired with a wrist‐worn device that recorded numerous data, as summarized in Table S1. The mobile app was designed specifically for the study by the Aparito team and the clinicians at the hospital. Some participants were provided with a smartphone to use if their own phone lacked the technical requirements for data transfer and Bluetooth required by the app. The app was paired with a wrist‐worn device (Hesvit S3, Hesvit, Shenzhen, Guangdong, China) using Bluetooth and participants were asked to wear the wrist‐worn device all the time apart from when washing, swimming, and charging the device.

#### Wrist‐worn device data

The wrist‐worn devices recorded the number of steps, heart rate (using an optical sensor), and minutes of light and deep sleep, calculating sleep duration and type from sensor data captured by a three‐axis acceleration sensor. The heart rate was derived from the green light sensor through photoplethysmography. For the sleep type, the company's proprietary algorithms used machine‐learning models validated against polysomnography to derive the sleep type from the tri‐axis accelerometer signals.

For data captured by the wrist‐worn device, days were partitioned at 12:00 to avoid subdividing the usual period for sleep. The proportion of wear time per day was calculated using the sleep and activity data. Heart rate was not consistently recorded by the wearable device owing to the device being worn in different positions or locations on the participants' upper limbs or the device not being worn tightly enough for the light sensor to be in contact with the skin. Therefore, heart rate data were not included in the analysis. A minute with no registered activity or sleep data was deemed non‐wear. A valid day was defined as having at least 10 hours of valid wear. Valid sleep data were determined as having sleep duration of more than 3 hours.^18^ A nap was defined as a sleep episode with a start time between the hours of 08:00 to 17:00.^18^ The wearable data available from the device were already classified by into activity type (walk/run) and sleep type (awake, deep sleep, light sleep) algorithms developed by Hesvit. Active time was defined as the time spent walking or running. Sedentary periods were defined as the time where participants were awake but not active. Sedentary, active, and sleep time were expressed as a percentage of total wear time for that day. Thereafter, percentage time spent in each of these categories was averaged between days.

#### Mobile app data

Caregivers of the participants were given instructions on how to use the app for reporting their child's seizures (including video capture), behavioural difficulties, poor sleep, illness, or any other events in real time. All participants' caregivers completed clinical interviews and self‐report questionnaires during initial assessments on their behalf. These included quality‐of‐life questionnaires such as the Child Health Utility 9D (CHU9D)^19^ and the EQ‐5D‐Y (child‐friendly version of the EQ‐5D‐3L)^20^ as well as customized questionnaires on medication adherence, ketogenic diet, and sleep and behavioural aspects. Each patient's ASMs with their individualized dose and frequency were explained during the baseline visit and the app was set up to provide alert notifications.

Continuous reminders were sent via the app at the time medication was due to support medication adherence. The caregivers were also able to indicate whether the medication was taken and explain the reason why if not. Changes in medication prescription were updated on the clinical dashboard to ensure the reminders sent reflected current medication regimens. A notification was displayed by the app when action was needed. A ‘Visits’ section captured all health‐care appointments. The CHU9D, EQ‐5D‐Y, ketogenic diet, and sleep questionnaires were set for completion every 30 days. A yes/no question was sent through the app every morning, allowing the caregivers to report their child's sleep quality. The medication adherence report was completed every 90 days.

The event option included a section to report any events, for example seizures or other events such as illness, behavioural issues, poor sleep, or stress in real time. The seizure option included a section for a real‐time report of seizure date and time, duration, type (clonic, tonic–clonic, spasms, myoclonic, absent, focal aware, focal unaware, described in non‐medical terms), ASM use, free text for further details, as well as possible video capture. Participants continued to attend routine care follow‐up visits at the clinic throughout the study period. Other details of this are reported in an earlier publication.^12^

### Clinical records data

Data extracted from clinical records are shown in Table S1. Clinical or psychosocial concerns raised at any epilepsy clinic for up to 2 years before starting the study were recorded. All participants' caregivers completed clinical interviews and self‐report questionnaires during initial assessments.

### Genetic data

#### Next‐generation gene panel sequencing

A customized next‐generation sequencing gene panel of 78 genes (listed in Table S2) was designed, comprising genes with established disease associations and clinical use, mainly in the developmental and epileptic encephalopathies. Saliva samples were obtained from each proband using Oragene saliva kits (DNA Genotek, Ottawa, Ontario, Canada), and DNA was extracted according to the manufacturer's instructions. Samples from parents, where available, were collected for potential segregation analysis. Next‐generation sequencing was on the Ion Torrent platform (Ion Torrent PGM, ThermoFisher Scientific, Waltham MA, USA); in addition, subsequent variant filtration and prioritization were performed as previously described.^14^, ^21^ Putative variants were selected for confirmation with direct cycle sequencing and segregation analysis using parental samples, where available. Confirmed variants were assigned pathogenicity classifications according to the guidelines of the American College of Medical Genetics and Genomics and the Association for Molecular Pathology.^22^

#### Pharmacogenomic data

A preliminary exploration of the pharmacogenomics of ASMs and drug‐resistant epilepsy in probands was undertaken to provide a possible direction for future pharmacogenomic research. This included both exploration of non‐specific absorption, distribution, metabolism, excretion, and toxicity variants and analysis of certain variants implicated in ASM metabolism in other populations. The VeriDose Core Panel, consisting of 73 target variants across 20 well‐known ADME genes—including 68 single/short nucleotide polymorphisms and five copy number variations, was chosen for the core panel analysis (Agena Bioscience, San Diego, CA, USA) (Table S3). In addition, a custom mass array containing variants in genes known to influence ASM metabolism in non‐African populations was designed using the online database PharmGKB (http://pharmgkb.org) (Table S4).

### Data analysis

#### Clinical and technology‐based data analysis

Descriptive statistics were used to analyse demographic variables. Normality of the variables was tested using a Shapiro–Wilk test. Differences in the number of seizures per month reported in clinical records compared with those reported using the technology were assessed using a Wilcoxon signed‐rank test (comparing paired quantitative data). Mann–Whitney *U* tests compared the participants' wearable outcomes with expected outcomes for typically developing peers of the same age range, as data were either not normally distributed or not continuous.^23^ A paired *t*‐test was used to compare the sleep duration recorded by the wearable device with the sleep duration reported by the caregivers, as data were paired and normally distributed. A Kruskal–Wallis test was used if comparisons involved more than two groups, especially for analyses of sleep data. Significance levels were set at *p* < 0.05. All analyses were conducted with R (https://www.R‐project.org/; R Foundation for Statistical Computing, Vienna, Austria).

#### Genomic and pharmacogenomic data analysis

For data from the next‐generation sequencing panel, confirmed variants following variant filtration and prioritization were assigned pathogenicity classifications according to the guidelines of the American College of Medical Genetics and Genomics,^22^ after being confirmed with direct cycle sequencing and segregation analysis. The genotype counts for each variant underwent statistical analysis for Hardy–Weinberg disequilibrium and a Pearson *χ* ^2^ test, using R software (version 4.1.1) and data for African reference populations available in the online database Ensembl (http://www.ensembl.org).

## RESULTS

### Demographic and clinical data

Thirty‐nine patients completed the study. One participant voluntarily withdrew from the mHealth aspect of the study because they felt overwhelmed with the devices. Inclusion was driven by parental acceptance rather than clinical phenotype, as more children met the inclusion criteria but parents did not provide consent for study participation and data collection. The study process, participant inclusion, and data capturing are illustrated in Figure S1 to help visualize the process. The median age of participants was 10 years (range 4–16) and 20 participants were female. Most participants (*n* = 27) self‐identified as Indigenous Black African (Table 1). Although structural brain abnormalities were common (41.0%), aetiology was unknown for many despite extensive investigations (35.9%). Five individuals (12.8%) had a probable genetic cause for their seizures identified although two variants remained classified as being of uncertain significance. Most children (*n* = 36, 92.3%) had neurodevelopmental or neurocognitive impairment. Only 23 were ambulator‐independent on the Functional Ambulation Category score, with eight children unable to walk.

**TABLE 1:** Participants' characteristics.

Characteristics	Whole study population (*n* = 39)		Participants included in seizures analyses during the study over 6 months (*n* = 21)	
	*n*	%	*n*	%
Age group at recruitment, years				
4–5	7	17.9	3	14.3
6–11	22	56.4	12	57.1
12–16	10	25.6	6	28.6
Sex				
Male	19	48.7	10	47.6
Female	20	51.3	11	52.4
Ethnicity				
Black Indigenous African	27	69.2	12	57.1
Mixed ancestry	10	25.6	8	38.1
European ancestry	1	2.6	1	4.8
Indian ancestry	1	2.6	0	0
Probable seizure aetiology^a^				
Genetic	3	7.8	3	14.3
Possible genetic	2	5.2	2	9.5
Infectious	4	10.3	3	14.3
Structural	16	41.0	4	19.0
Unknown	14	35.9	7	33.3
Age at onset of epilepsy, months				
Median (Q1–Q3)	29.0 (8.0–60.0)	NA	48.0 (7.0–60.0)	NA
Range	0.03–126.0	NA	0.03–114.0	NA
History of status epilepticus				
Yes	16	41.0	6	28.6
No	23	59.0	15	71.4
Family history of seizures				
Yes	10	25.6	6	28.6
No	29	74.4	15	71.4
Neurocognitive and neurodevelopmental impairment				
Cognitive impairment	30	76.9	17	81.0
Global developmental delay	6	15.4	2	9.5
No neurodevelopmental or neurocognitive impairment	3	7.7	2	9.5
Number of ASMs at baseline				
2	8	20.5	3	14.3
3	27	69.2	16	76.2
>3	4	10.3	2	9.5
Number of ASMs trialled previously				
2	3	7.7	0	0.0
3	9	23.1	4	19.0
>3	27	69.2	17	81.0
Non‐adherence history				
Yes	14	35.9	7	33.3
No	25	64.1	14	66.7

^a^ Note that participants could present with multiple neurological conditions (conditions under other included).

Abbreviation: ASM, antiseizure medication.

### Engagement with wearable devices

Engagement with the wearable devices was reported by Davies et al.^12^ A total of 37 out of 39 participants had valid wearable days. All had valid sleep data for at least 1 day of the study. The participants produced valid days for a median of 50 days (IQR 35–74, range 1–185) and valid sleep data for a median of 43 days (IQR 28–60, range 1–182). Participants wore the wearable device for a median of 17.9 hours (IQR 17.1–18.5) each day (range 13.8–20.2). During the first month of remote monitoring, 35 participants (94.6%) wore the device for a median of 20 days (IQR 9–26, range 1–30) and a median of 18.0 hours (IQR 17.0–19.1, range 11.7–21.6) each day. At 6 months, 22 out of 37 participants (59.5%) were still using the wearable device, with a median use of 8 days (IQR 4–18) and a median wear time of 16.8 hours each day (IQR 15.9–17.6). The 20 patients who wore the device during both the first month and the sixth month showed a decrease in median use (20 days [IQR 10] vs 8 days [IQR 13.3] respectively, *p* = 0.02, Wilcoxon signed‐rank test). Their wear time remained consistent with a median of 17.3 hours each day (IQR 16.4–19.0) during the first month and 16.8 hours (IQR 15.9–17.7) during the sixth month (*p* = 0.81, Wilcoxon signed‐rank test).

### Seizure reporting

Previous and consecutive clinic documentation of seizures was compared with app findings. We found a statistically significant discrepancy in the median number of seizures per month recorded by the mobile application, with 2.0 seizures per month using the app compared with 8.0 per month in clinic records in the 2 years before the study (*n* = 28, *p* < 0.001, *r* = 0.64, 95% confidence interval [CI] 2.62–14.25, Wilcoxon signed‐rank test). A similar difference was observed between the median 2.0 seizures per month reported using the app and the 5.5 seizures per month reported in clinic records during the study period (*n* = 21, *p* < 0.001, *r* = 0.76, 95% CI 2.25–15.75, Wilcoxon signed‐rank test). The median seizure durations reported via the app during the study were comparable to those reported in the clinic in the 2 years before the study (*n* = 24, *p* = 0.94, *r* = 0.04, 95% CI −1.50 to 3.00, Wilcoxon signed‐rank test) and during the study (*n* = 11, *p* = 0.19, *r* = 0.34, 95% CI −1.75 to 14.37, Wilcoxon signed‐rank test) as shown in Table 2. While the sample size had decreased for the comparisons of both records and app data, the summary statistics were similar for both median seizure frequency and duration for the total sample and those subsets included in analyses with both clinical record and app report data. During the 2 years before the study, the median number of clinic visits was six (IQR 5–7). The median percentage of visits where seizure frequency was recorded was 72.7% (IQR 50.0–84.5), while this was 33.3% for seizure duration (IQR 16.7–46.4). In comparison, there were two (IQR 1–2) clinic visits for the 6 months during the study, with the median percentage of visits that recorded seizure frequency and seizure duration being 100% (IQR 50.0–100.0) and 33.3% (IQR 0.0–50.0) respectively.

**TABLE 2:** Seizure reporting in medical records and app reports for the study population.

Measure/test	Medical records before study (2 years), *n* = 28	Medical records during the study (6 months), *n* = 21	App data during the study (6 months), *n* = 28	Comparison measure	App compared with medical records before study	App compared with medical records during study
**Number of reported seizures per month**				**Wilcoxon signed‐rank test**		
Sample size	28	21	28	Sample size	*n* = 28; 28	*n* = 21; 21
Median	8.0	5.5	2.0	*p*	<0.001	<0.001
IQR	2.5–15.2	2.0–15.5	1.4–3.1	Effect size	*r* = 0.64	*r* = 0.76
Range	0.5–67.5	0.3–52.5	1.0–9.0	95% CI	2.62–14.25	2.25–15.75
**Median seizure duration in minutes**				**Wilcoxon signed‐rank test**		
Sample size	24	11	24	Sample size	*n* = 24; 24	*n* = 11; 11
Median	2.4	3.0	2.0	*p*	0.94	0.19
IQR	1.5–5.1	1.0–7.5	1.0–5.0	Effect size	*r* = 0.04	*r* = 0.34
Range	0.08–20.0	0.02–33.7	1.0–12.5	95% CI	−1.50 to 3.00	−1.75 to 14.37

*Note:* Seizure diaries kept in the 3 months before the start of the study were not well kept or returned and thus not included in analyses/this table.

Abbreviations: CI, confidence interval; IQR, interquartile range (Q1–Q3).

**FIGURE 1:**
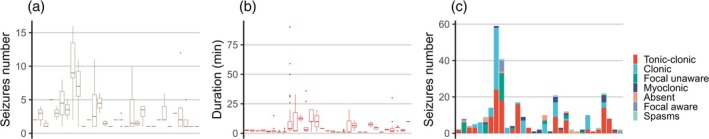
Reported seizures via the app. Across all panels, a box represents the data of the same participant. (a) Boxplot of the number of seizures per month. (b) Boxplot of the seizure duration for each participant. (c) Number of seizures by type.

In total, 287 seizures were reported by 31 participants using the app. On average, each participant recorded 2.7 seizures per month (SD 2.0), with individual seizure numbers varying from 1 to 16 per month (Figure 1a). The seizures lasted 4.7 minutes (SD 3.9) and individual duration ranged from 1 to 90 minutes (Figure 1b). The most common seizure types experienced by the participants were tonic–clonic (126 seizures, 43.9%), tonic (84, 29.3%), and focal unaware (32, 11.1%) (Figure 1c). In comparison, when using paper‐based diaries to log seizures during the run up to the study, only two (5.1%) participants returned diaries where seizure occurrences were marked with only a tick.

### Activity and sleep data

Despite more than half of participants being independent ambulators, the median activity level as measured by steps for the cohort (4257 steps, IQR 3213–6118) was almost a third below the age‐matched typically developing peers (*p* < 0.001, *r* = 0.86, 95% CI 7107–8789, Welch's *t‐*test) (Figure S2). Other comparisons between participants for activity (sedentary and active time) according to sex, age group, formal/informal housing, average seizures per month, ambulation status, and reported mobility issues are shown in Figure S3.

Among the 37 participants who used the wearable device, the average sleep duration was 7.6 hours (SD 1.2). The participants spent on average 6.5 hours (SD 0.8) in nocturnal sleep and a median 1.0 hours (IQR 0.7–1.3) in daytime naps. The participants spent a median 94.0% (IQR 91.2–96.1) of nocturnal sleep time in light sleep, equivalent to 5.9 hours (SD 0.6), and a median 6.0% (IQR 3.9–8.8) in deep sleep, amounting to a median 0.4 hours (IQR 0.2–0.8). During naps, the proportions of light and deep sleep were on average 86.0% (SD 8.1) and 14.0% (SD 8.1) respectively.

Ten participants (25.6%) reported experiencing sleep issues in their routine clinic appointments at least once in the 2 years before the study. Their average nocturnal sleep duration during the study was 6.5 hours and their median nap duration was 1.0 hours, which were similar to those of participants who did not report any pre‐study sleep issues (*p* = 0.87, *r* = 0.04, 95% CI −0.41 to 0.49, independent *t*‐test, and *p* = 0.45, *r* = 0.13, 95% CI −0.58 to 0.23, Mann–Whitney *U* test respectively).

We found no differences between participants grouped by sex, age, type of housing, bed sharing, average seizure frequency, and reported sleep issues and duration (Figure S4).

**FIGURE 2:**
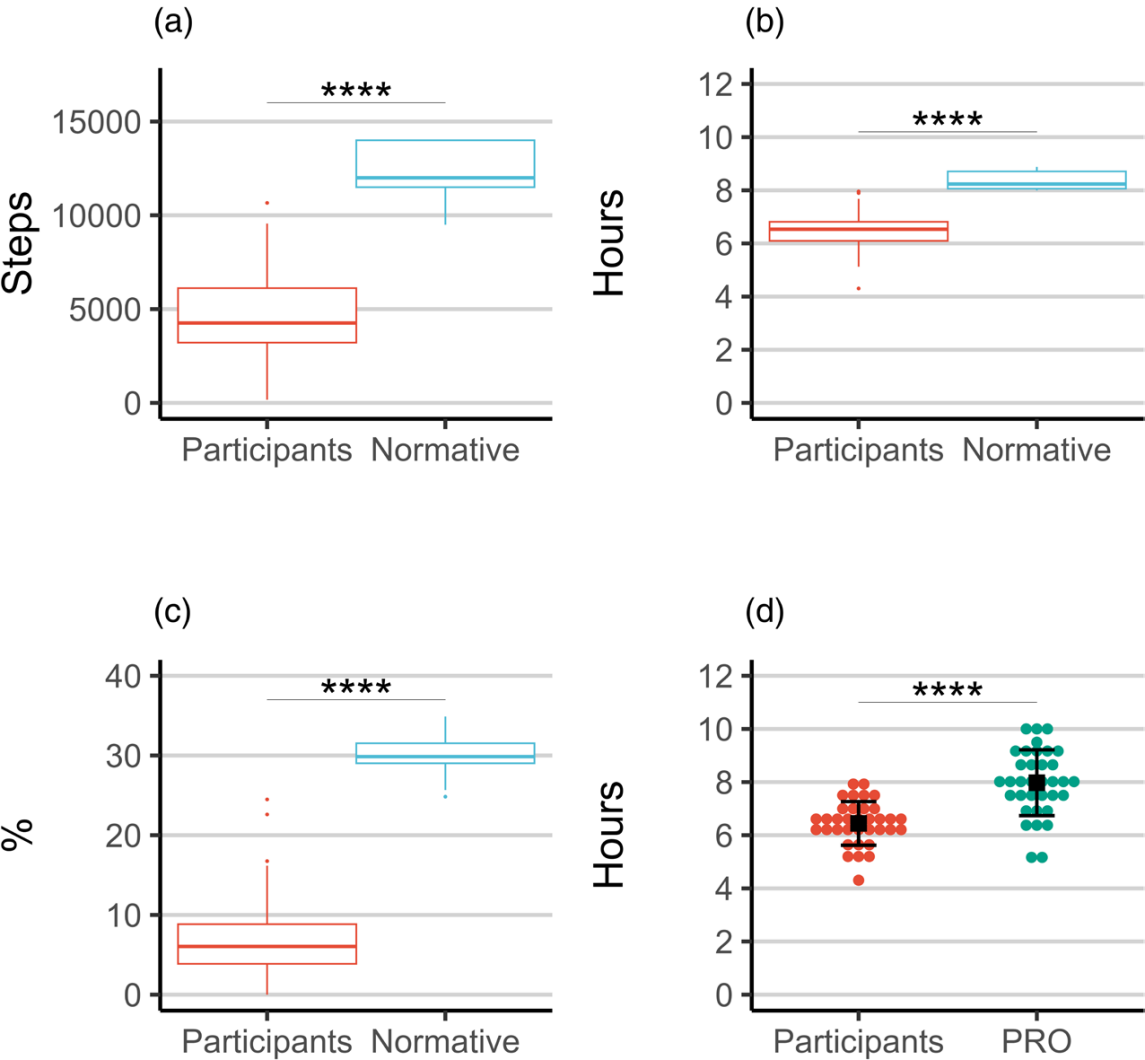
Distribution of (a) daily steps, (b) nocturnal sleep duration, and (c) proportion of deep sleep in the participants and typically developing peers. (d) Distribution of nocturnal sleep duration as recorded by the wearable device and self‐reported by the caregivers (mobile patient reported outcomes; mPROs); mean and standard deviation are shown in black. Significance levels for the Welch's *t*‐tests (comparing participants with typically developing peers) and paired *t*‐tests (comparing participants' sleep captured via the wearable device and in the patient‐reported outcomes) are shown on top (****p < 0.001).

Compared with expected sleep duration for age‐matched typically developing peers,^24^ the participants had a shorter median nocturnal sleep duration, with 8.2 hours (IQR 8.1–8.7) in typically developing peers compared with 6.5 hours (IQR 6.1–6.8) in participants (*p* < 0.001, *r* = 0.86, 95% CI 1.60–2.10, Mann–Whitney *U* test) (Figure 2b). Participants also showed a lower median proportion of sleep spent in deep sleep, with 29.9% (IQR 29.0–31.5) for typically developing peers compared with 6.0% (IQR 3.9–8.8) for participants (*p* < 0.001, *r* = 0.86, 95% CI 21.60–25.13, Mann–Whitney *U* test) (Figure 2c). Subjective self‐report of sleep duration by the caregivers was found to be longer than the nocturnal sleep duration recorded by the wearable device, with 8.0 hours (SD 1.3) reported and 6.4 hours (SD 0.8) recorded (*n* = 35, *p* < 0.001, *r* = 1.44, 95% CI 1.06–2.00, paired *t*‐test) (Figure 2d).

### Self‐ or proxy‐reported quality of life via mobile patient‐reported outcomes

The results on self‐ or proxy‐reported quality of life data are given in Appendix S1, including self‐reported sleep and medication adherence, as well as quality of life (Tables S5 and S6).

### Genetic results

#### Next‐generation sequencing panel results

Following variant filtration and prioritization, 17 were selected for confirmation and further investigation (Figure S5). Of these, direct cycle sequencing confirmed putative variants in 12 probands (one variant each).

Five of the 12 confirmed de novo variants in autosomal dominant genes, as shown by segregation analysis using parental DNA available. Variants inherited from an unaffected parent were excluded from further analysis.

Two probands were found to have pathogenic *SCN1A* variants and one had a likely pathogenic variant in *GRIN2A*. Two probands had variants of uncertain significance in *GABRG2* and *GRIN2B* respectively (Table 3). The two pathogenic *SCN1A* variants were also reported by Esterhuizen et al.^15^

**TABLE 3:** Relevant variants identified on next‐generation sequencing.

Gene (NCBI RefSeq)	rs ID and ClinVar ID	Variant type	Coding change (hg19 genomic coordinate)	Amino acid change	Zygosity	Inheritance	ACMG classification (ACMG criteria)	Clinical presentation
*SCN1A* (NM_006920.5)	rs121918624 ClinVar ID:12889	Nonsense	c.664C>T (chr2: 166909392)	p.Arg222Ter	Heterozygous	De novo	Pathogenic: PVS1, PS2, PM2, PP4, PP5	Focal seizures evolved to multiple seizure presentations; neuroregression
*SCN1A* (NM_006920.5)	rs1553521567 ClinVar ID: 530456	Splice acceptor	c.4444‐1C>T (chr2:166852628)	N/A	Heterozygous	Not established	Pathogenic: PVS1, PM2, PP4, PP5	Multiple seizure presentations; intellectual disability
*GRIN2A* (NM_001134407.2)	rs796052549 ClinVar ID:205657	Missense	c.2191G>A (chr16:9892299)	p.Asp731Asn	Heterozygous	Not established	Likely pathogenic: PM2, PP2, PP3, PM5	Focal seizures; intellectual disability
*GABRG2* (NM_198903.2)	N/A	Missense	c.211A>G (chr5:161520937)	p.Asn71Asp	Heterozygous	Not established	Variant of uncertain significance: PM2, PP2	Left hemiplegia; neuroregression; multiple seizure presentations
*GRIN2B* (NM_000834.4)	rs1042339 ClinVar ID: 245684	Missense	c.3499G>A (chr12:13716673)	p.Val1167Ile	Heterozygous	Not established	Variant of uncertain significance: PM2, P2, PP4	General tonic–clonic seizures progressed to multiple seizure presentations neuroregression

ACMG criteria:^22^ PVS1 (pathogenic very strong), nonsense variant; PS2 (pathogenic strong), de novo mutation; PM2 (pathogenic moderate), low population frequency; PP4 (pathogenic supporting), expected phenotype; PP5 (pathogenic supporting), described as pathogenic in online databases; PP2 (pathogenic supporting), missense variant in a gene that has a low rate of benign missense variation and in which missense variants are a common mechanism of disease; PP3 (pathogenic supporting), computational evidence for pathogenicity; PM5 (pathogenic moderate), a different missense variant at the same site is pathogenic.

Abbreviations: ACMG, American College of Medical Genetics and Genomics; ID, identifier; N/A, not available; NCBI, National Center for Biotechnology Information; rs, reference single nucleotide polymorphism cluster.

#### Pharmacogenetic array results

After stratification of the cohort on the basis of ethnicity, only the group of Indigenous Black African ancestry was of sufficient size (*n* = 27) for inclusion in the Pearson *χ* ^2^ test; thus results were limited to this group. Of the 73 known ADME variants in the Veridose Core Panel, only 30 could be analysed (Table S7). The remaining 38 were represented by a single genotype, and thus were excluded from analysis. Of the variants that could undergo statistical analysis, one, *CYP2D6* rs59421388, seemed to be trending towards significance (*p* = 0.05). Two of the eight variants in the custom array were out of Hardy–Weinberg equilibrium (*HTR2C* rs1414334 and *SCN1A* rs3812718). In addition, *EPHX1* rs1051740, which is associated with the metabolism of carbamazepine in East Asian and European populations, seemed to be significantly differently distributed in our cohort compared with the published data from the general African population (*p* = 0.02) (http://www.ensembl.org). Two variants may be worth exploring in future analyses, namely *EPHX1* rs2234922 (*p* = 0.08) and *SCN1A* rs3812718 (*p* = 0.05), both associated with the metabolism of phenytoin in East Asian and European populations (Table S8).

## DISCUSSION

This pilot study evaluated the potential implementation of mHealth and genetic analysis as precision medicine initiatives in a South African public health‐care service for children with drug‐resistant epilepsy. mHealth technology captures important clinical data. While there are serious challenges to implementing mHealth and other precision health technologies in LMICs, this study and the previous paper on feasibility^12^ have shown value in such technology and precision medicine being used in health‐resource‐limited settings.

Although structural, infectious, and vascular aetiologies, or a combination of these, may be more common than in higher‐income countries,^5^ genetic aetiology remains as prevalent as elsewhere and has direct relevance to care.^5^, ^14^, ^15^ For the high proportion of cases for which no genetic aetiology was identified, next‐generation sequencing analysis was undertaken using a panel design aimed at the developmental epileptic encephalopathies, and its sensitivity in this context was probably suboptimal. Additional testing using whole‐exome or whole‐genome sequencing is likely to yield more informative findings and resolve more cases in genes excluded from the panel.^25^

One of the most significant benefits anticipated of mHealth is the accuracy of seizure recall over extended time periods. Fewer seizures were recorded using the app (2.0 seizures per month) compared with clinic records in the 2 years before the study (8.0 seizures per month) and with clinic records during the study period (5.5 seizures per month). Interestingly, this contrasts with a previous finding of this cohort, where seizure reporting via the app was significantly higher than in the paper‐based diary (79% compared with 5%). However, those previous findings did not account for reported seizure frequency, and before the start of the study seizure frequency was only noted in 72.7% of clinical notes at clinic visits. Further, more in‐depth detail of seizure events (e.g. type and duration) was provided via the app than during clinic visits. This further alludes to discrepancies in memorization and recall of seizure elements in patients, making accurate clinical assessments difficult.^13^ Some bias may have been introduced during clinic visits as clinicians were aware of the app being in place which may have influenced clinical data capturing during visits. However, the potential for user error or technical issues may also have to be considered, although this was not a finding reported in the previously published data of this study.^12^

A significant finding was the advantage of mHealth recording other lifestyle parameters that may not be noticed in busy clinics. Physical activity and sleep quality, as well as mood and behaviour, are important but neglected factors and have a bidirectional relationship with epilepsy.^26^, ^27^ Patients with epilepsy are less physically active than the general population, which was also shown in this study, despite physical activity showing benefits for psychosocial well‐being and mitigating seizures.^28^ The exclusion of heart rate data because of inconsistent recording by the wearable device was a limitation, given that heart rate can be a critical parameter in understanding seizure activity and general health. Sleep recording and caregiver reports in this study showed significant sleep disturbances, with the average nocturnal sleep duration being almost 2 hours less than that of typically developing children (6.3 hours vs 8.4 hours), and deep sleep constituting a much lower proportion of sleep (7.8%) compared with typically developing populations (30.1%).^24^ This important observation would be difficult to elicit in a clinic setting given that caregivers reported a much higher sleep duration (8.0 hours) than that recorded by the wearable device, even though almost a third reported poor sleep using the mobile patient‐reported outcomes, ranging from 0% to 80%, mostly related to anxiety, pain, and restlessness. Discrepancies may also be related to irregular wearing of the devices or lack of awareness of sleep disturbances.^12^ These findings further add to research highlighting the role of monitoring and regular prompted caregiver reports in eliciting sleep issues and other health‐related activities.^27^ Furthermore, a positive outlook may skew reporting of events to health providers at clinic appointments, as indicated by the very low values for the CHU9D and EQ‐5D‐Y, where caregivers of affected children indicated that they had ‘no problem’ in four out of a total five dimensions, described previously.^12^ Recording these parameters may be useful to assess seizure control and quality of life, and both pharmacological and non‐pharmacological management. The data should enable the clinician to move beyond the typical concise and targeted clinical assessment that occurs in the clinic setting.

Furthermore, it is important to include data from other groups of patients outside Western or resource‐equipped countries usually used to develop digital health models and platforms, as this prevents unidimensional or uniform modelling data which have been shown to lead to bias in digital health‐care technology.^29^ Previous studies have shown utility and feasibility, as well as acceptability, in implementing these digital health approaches in African contexts and LMICs in general.^29^ In SSA, including South Africa, mHealth has already been applied for various purposes, such as ordering medications, and not only younger age groups have shown enthusiasm—older age groups between 46 and 60 years have been engaged in, and recognized the value of, digital health solutions.^30^ Combining increasingly available mobile technology and digital health‐care applications has the potential to dramatically improve health‐care outcomes for millions of people, from access to diagnosis and treatment.

Two pathogenic truncating *SCN1A* variants were identified in respective probands, one likely pathogenic missense variant was found in *GRIN2A*, and two missense variants of unknown significance were found in *GABRG2* and *GRIN2B* (Table 3). For the patients with *SCN1A* variants, this finding enabled clinicians to focus on targeted ASM.

The next‐generation sequencing panel demonstrated a diagnostic yield of 7.5%. While this is low compared with the diagnostic yields of up to 50% reported in the literature,^31^, ^32^ some previous studies have also reported lower yields.^11^ It is important to consider that only 40 participants were tested, that the study inclusion criteria were broad (older than 4 years and drug‐resistant epilepsy), and that there was a high frequency of other aetiologies of epilepsy. However, this low yield may also indicate limitations of the gene panel used, suggesting a possible need for more comprehensive genomic approaches, such as whole‐exome or whole‐genome sequencing.^25^ Nonetheless, the financial costs of such comprehensive approaches may limit the potential benefits.

All the identified variants occurred in autosomal dominant genes. This was expected as, according to current knowledge, monogenic epilepsies are most commonly associated with de novo variants in autosomal dominant genes, which may occur independently of genetic variation between populations.^33^ This may not be the case with recessive variants, which may be population‐specific or prevalent. Therefore, future research may reveal other, more locally relevant genes. Additional genetic research will also facilitate variant interpretation, as better representation of the African population and its genetic variation in the genome browsers will help to implicate or exclude many of the currently reported variants of uncertain significance in African patients.

Lastly, the pharmacogenomics aspect included investigation of known absorption, distribution, metabolism, excretion, and toxicity variants. Of the other 38 variants analysed, several were of interest and two were potential candidate gene markers. *CYP2D6* (rs59421388), a variant for which little previous research is available, seemed to be trending towards significance (*p* = 0.05). Two variants for *EPHX1* may be worth investigating further: the distribution of *EPHX1* rs1051740 in our group seemed to be significantly different to the general African population, while the difference in distribution of *EPHX1* rs2234922 was trending towards significance. Both variants have been implicated in carbamazepine metabolism in non‐African populations.^34^, ^35^ Lastly, *SCN1A* rs3812718 seemed to be trending towards significance (*p* = 0.05), and has been associated with phenytoin metabolism and carbamazepine resistance in non‐African populations.^36^ *SCN1A* is one of the most frequently identified causative genes in the developmental epileptic encephalopathies.^15^, ^25^ However, these findings in particular should be interpreted cautiously as they are only trending towards significance and are only preliminary results and not definitive.

Despite significant health benefits, there were considerable barriers to the implementation of this mHealth technology in this context.^12^, ^28^ Many participants were nervous about using the wearable device outside the home because of fear of theft or damage and personal safety, even at schools. Behavioural and cognitive factors, such as hyperactivity, intellectual disability, and others, further compromised the use of mHealth. A common issue was lack of technological knowledge and struggling to use smartphones and Bluetooth connection to upload data, partly because of poor access to networks and cost, especially in rural areas. The engagement with wearable devices decreased over time, with only 59.5% of participants still using the devices at 6 months. This drop‐off may have introduced bias, as the remaining participants may not have been representative of the entire cohort. It was difficult to address this attrition in the quantitative analyses other than by selecting the appropriate statistical tests for analyses given the decreased sample sizes. The reasons for decreased engagement were explored extensively and have been reported in the previously published paper.^12^ Resultant data issues such as the exclusion of heart rate data because of inconsistent recording by the wearable device was a significant limitation, given its importance in indicating seizure activity and general health. Underlying issues of technological literacy, device maintenance, and data privacy may further challenge scalability of mHealth in the context of SSA. Thus, contextual awareness and factoring in high prevalence of poverty in populations of patients is essential when implementing mHealth approaches, especially for future studies. This is imperative to support populations in LMICs and avoid exacerbating health inequalities as health care is becoming increasingly digitized on a global level. Other ethical considerations of using mHealth and genetic testing in this context also require further discussion and study, including issues about confidentiality, consent, accessibility of data, population/genetic diversity, and access to results of outcomes for study participants, as well as their applicability to other patients in a similar context in LMICs or countries in SSA, including risk–benefit analyses.

In addition, the potential biases introduced by clinicians being aware of the app may have influenced clinic consultations and thus clinical records, as possibly indicated by the change in the reported number of seizures per month during the study period compared with before. In future studies, it would be beneficial to blind clinicians to, or reduce awareness of, the introduction of an mHealth application before or during the study period to reduce risk of bias. A small degree of potential selection bias posed another limitation, as inclusion was driven by parental consent more than clinical phenotype. This might further limit the study's applicability to other populations or settings which may display different consent rates and reasons for non‐consent, especially if they allow for analysis of more specific clinical phenotypes. However, only 3 out of 50 participants approached initially declined participation (for fear of breaking the watch); thus it is unlikely that this bias would have a large impact. A greater issue may have been introduced by drop‐off bias given the decreased engagement over time, which could only be partly addressed in quantitative analyses by comparing the total and final samples. The causes and factors for this drop‐off were further explored in the previously published study.^12^

A major limitation of this study was the small sample size and decreased statistical power. This, in combination with the nature of the data, necessitated the use of non‐parametric tests, and increased the risk of type I and especially type II errors by reducing statistical power. These tests and the small sample size prevent us making inferences from these findings and limit the generalizability of the results and their applicability to broader populations, until more high‐powered, larger, and more comprehensive studies in the context of SSA confirm or refute them. Although multiple tests were conducted, these were to test different hypotheses and were very low in number, and significant findings showed *p*‐values less than 0.001; thus correction for multiple tests was not done and was unlikely to have influenced main findings.

Thirty‐eight pharmacogenetic variants could not be analysed because of completely uniform genotype distribution. It is possible that these variants simply do not exist in the African population, but it is more likely that the sample size of 40 probands was too small to detect variation. Future studies will also need to address the current issues of unproven replicability and lack of generalizability of studies applying personalized care and mHealth technology in patients with epilepsy.^37^ Incorporating more diverse populations and different contexts may play a role in addressing these problems.^38^ Future studies may benefit from guidance by frameworks for validation that include standardization of relevant data tailored both for developers and for clinicians.^39^ Such frameworks or guidelines would ideally address performance, explainability, generalizability, replicability, and clinical utility, as well as enable accessibility by using open‐source tools.^37^, ^40^

While research in this field in SSA is still in its early stages, this study, in combination with further research, may inform both policy and clinical practice in SSA in the field of treatment‐resistant epilepsy by informing gene panel designs for diagnostic testing and influencing treatment decisions. The study contributes to important research in developing population‐specific reference databases to improve the interpretation of genetic variants in Africa. These results also indicate potential pitfalls in implementing personalized medicine in resource‐limited contexts, both in the form of genetic testing and mHealth technology.

## CONCLUSION

This study demonstrated that mHealth in epilepsy, while possible in resource‐limited settings, is limited by contextual psychosocial challenges. Nonetheless, valuable insights into seizure frequency, duration, and semiology may be gained by digital health approaches. Furthermore, the identified potential candidate gene markers in ASM metabolism may be relevant to the local population and may present a catalyst for future pharmacogenomic research in African patients with epilepsy, although applicability to broader populations may be limited by the small sample size. There is a significant need for global health and personalized medicine for research in diverse and resource‐limited settings. This study provided a more comprehensive view of patients in this setting by integrating data from clinical records, wearable devices, and genetic analysis, thereby capturing more accurate and detailed data on seizure activity, sleep, physical activity, and genetic variants. Expanding such innovations in diverse settings can drive more equitable health‐care delivery, paving the way for more inclusive and effective solutions in global health.

## FUNDING INFORMATION

The Newton Fund; South African Medical Research Council (SAMRC); the National Research Foundation (NRF) of South Africa. The research reported in this publication was supported by the Strategic Health Innovation Partnerships (SHIP) Unit of the South African Medical Research Council with funds received from the South African Department of Science and Technology.

## CONFLICT OF INTEREST STATEMENT

EHD is both a shareholder and employee at Aparito; SK is an employee at Aparito. The other authors have stated that they had no interests that might be perceived as posing a conflict or bias.

## Supporting information


**Figure S1:** Flow chart of participants and study process.

**Figure S2:** Box plots of average activity level as measured by steps for the cohort (red) compared to age‐matched controls (blue).

**Figure S3:** Box plots of wear time spent in sedentary behaviour or physical activity for participants

**Figure S4:** Box plots of time spent in nocturnal sleep or daytime nap for participants

**Figure S5:** Flow diagram outlining the process of variant prioritization, confirmation, and interpretation drop‐off of probands.

**Table S1:** mHealth technology‐based and clinical records data collected.

**Table S2:** Genes incorporated in gene panel design.

**Table S3:** The 73 target variants included in The VeriDose® Core Panel.

**Table S4:** Variants and respective genes used in custom mass array designed using the online database PharmGKB to identify variants and genes known to influence ASM metabolism in non‐African populations.

**Table S5:** Proportion of levels of CHU9D by CHU9D dimension and by time of assessment.

**Table S6:** Proportion of levels 1, 2 and 3 by EQ‐5D‐Y dimension and by time of assessment and EQ VAS by time of assessment.

**Table S7:** General pharmacogenomic (ADME) variant (*n* = 30) genotype counts, Hardy–Weinberg equilibrium P‐value, and Pearson's Chi‐squared test P‐value.

**Table S8:** ASM‐specific variant (*n* = 8) genotype counts, Hardy–Weinberg equilibrium (HWE) p‐value, and Pearson's Chi‐squared test p‐Value.

**Appendix S1:** Supplementary results

## Data Availability

The data that support the findings of this study are available from the corresponding author upon reasonable request.
