# Supplementary figures and images for: Personalized care of paediatric drug‐resistant epilepsy in Africa: A single‐centre pilot study utilizing mobile health and genetic testing
Source: Dev Med Child Neurol. 2025 Aug 20;68(3):394–406. doi: 10.1111/dmcn.16478 (PMC12875146; doi:10.1111/dmcn.16478)

**
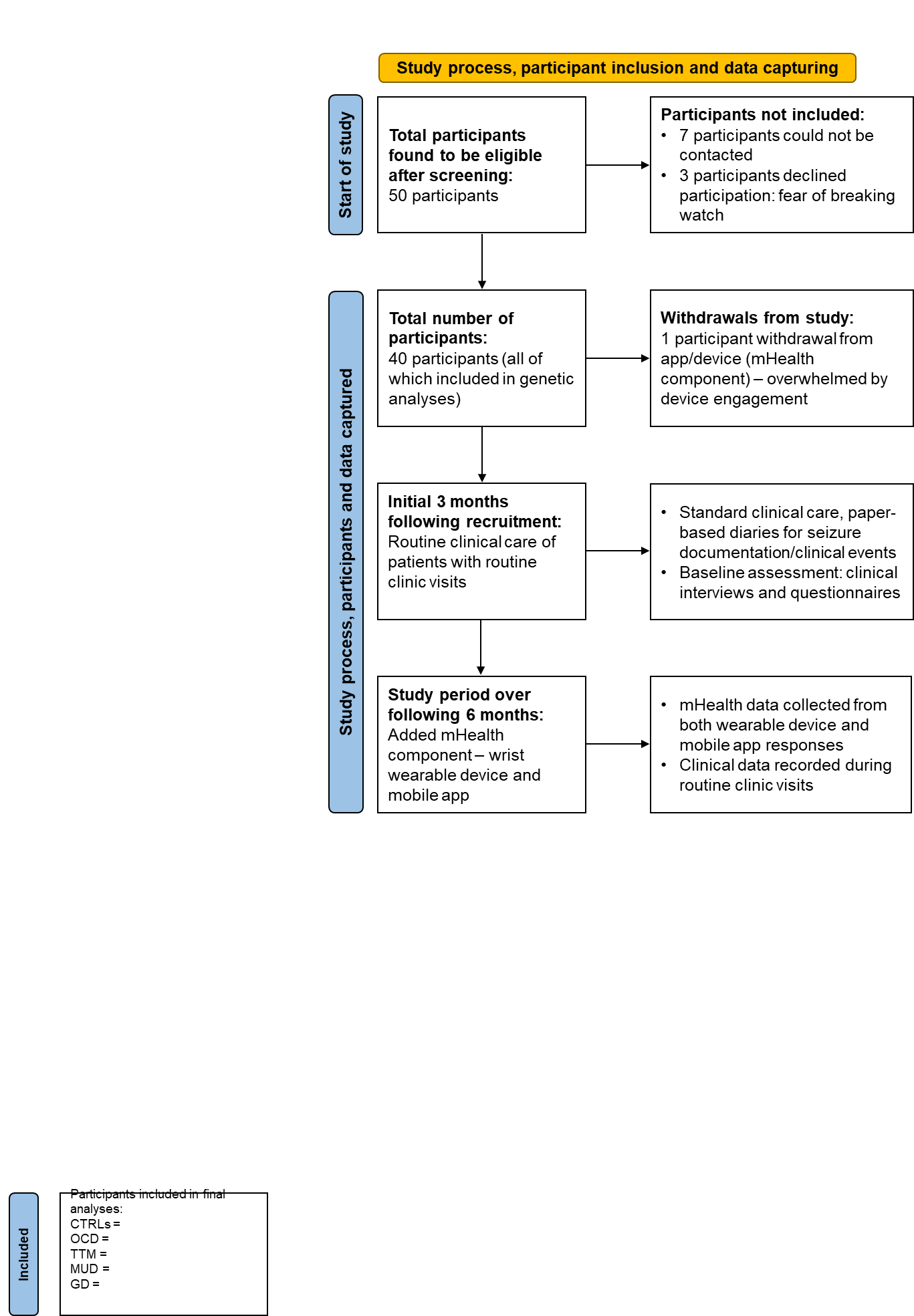
Figure S1: Flow chart of participants and study process**

Supplement: Supplementary file 1 — Figure S1: Flow chart of participants and study process. [file DMCN-68-394-s012.docx]

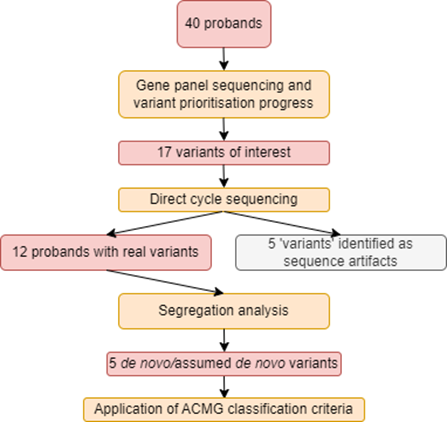

Supplement: Supplementary file 5 — Figure S5: Flow diagram outlining the process of variant prioritization, confirmation, and interpretation drop‐off of probands. [file DMCN-68-394-s006.png]

**Supplementary Table S3:** The 73 target variants included in The VeriDose® Core Panel


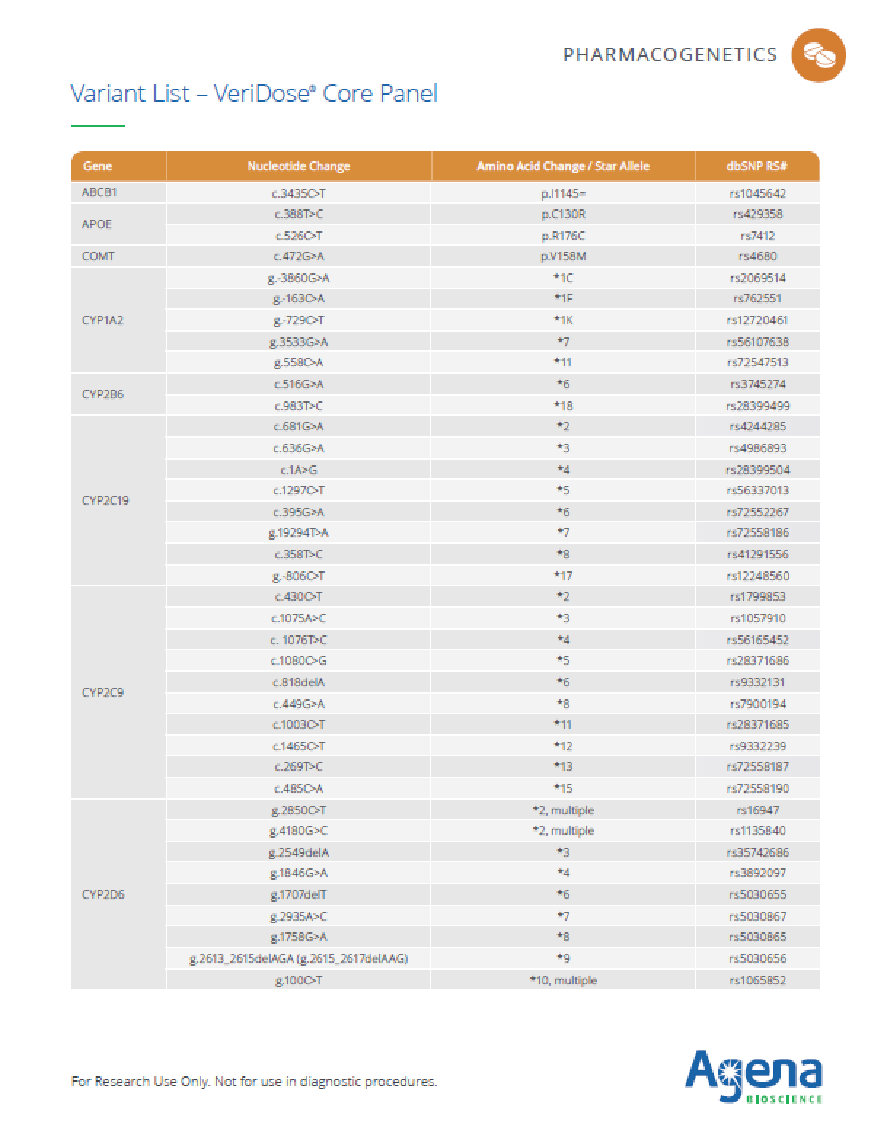


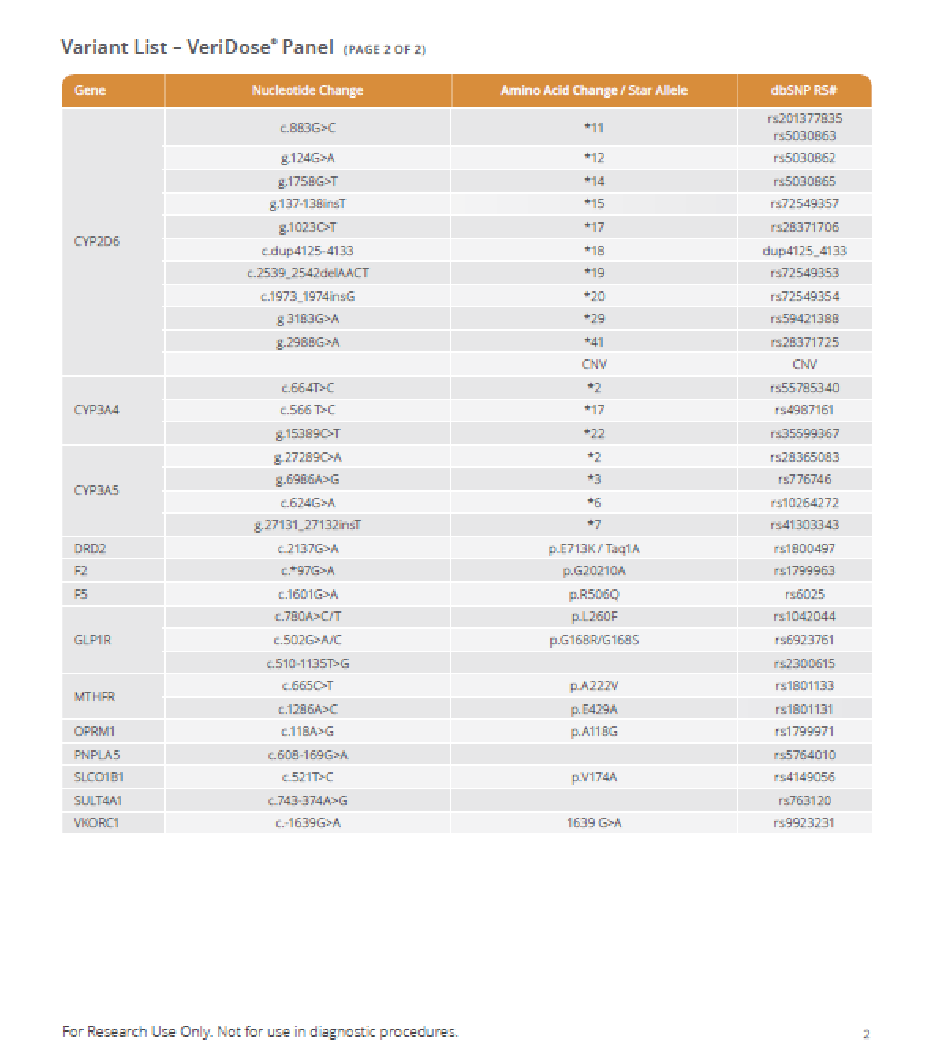

Supplement: Supplementary file 8 — Table S3: The 73 target variants included in The VeriDose® Core Panel. [file DMCN-68-394-s011.docx]
