# Supplementary material for: Personalized care of paediatric drug‐resistant epilepsy in Africa: A single‐centre pilot study utilizing mobile health and genetic testing
Source: Dev Med Child Neurol. 2025 Aug 20;68(3):394–406. doi: 10.1111/dmcn.16478 (PMC12875146; doi:10.1111/dmcn.16478)
Supplement: Supplementary file 2 — Figure S2: Box plots of average activity level as measured by steps for the cohort (red) compared to age‐matched controls (blue). [file DMCN-68-394-s014.docx]

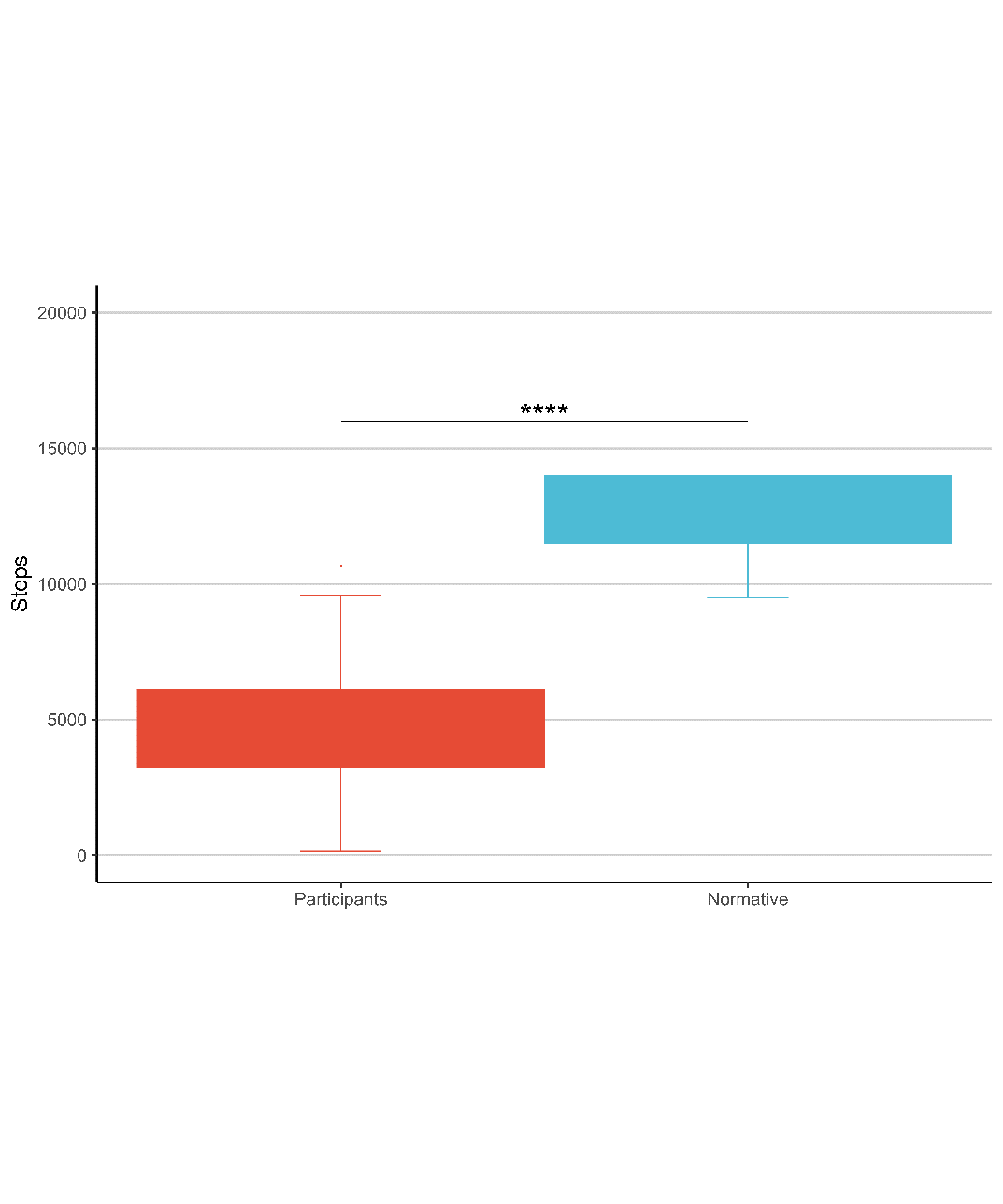


**Figure S2.** Box plots of average activity level as measured by steps for the cohort (red) compared to age-matched controls (blue). Despite more than half of participants being independent ambulators, the average activity level as measured by steps for the cohort (4539, SD 2495) was almost a third below age matched controls (p<0.0001, Mann-Whitney test). The box-plots show, from bottom to top, the 25th percentile, median, and 75th percentile values, and the error bars indicate the lower and upper adjacent values. The upper adjacent value is defined as the largest data point ≤75th percentile + 1.5 interquartile range (IQR), and the lower adjacent value is defined as the smallest data point ≥25th percentile – 1.5 IQR.
