# Supplementary material for: Personalized care of paediatric drug‐resistant epilepsy in Africa: A single‐centre pilot study utilizing mobile health and genetic testing
Source: Dev Med Child Neurol. 2025 Aug 20;68(3):394–406. doi: 10.1111/dmcn.16478 (PMC12875146; doi:10.1111/dmcn.16478)
Supplement: Supplementary file 3 — Figure S3: Box plots of wear time spent in sedentary behaviour or physical activity for participants [file DMCN-68-394-s013.docx]

**Supplementary Figure S3.**


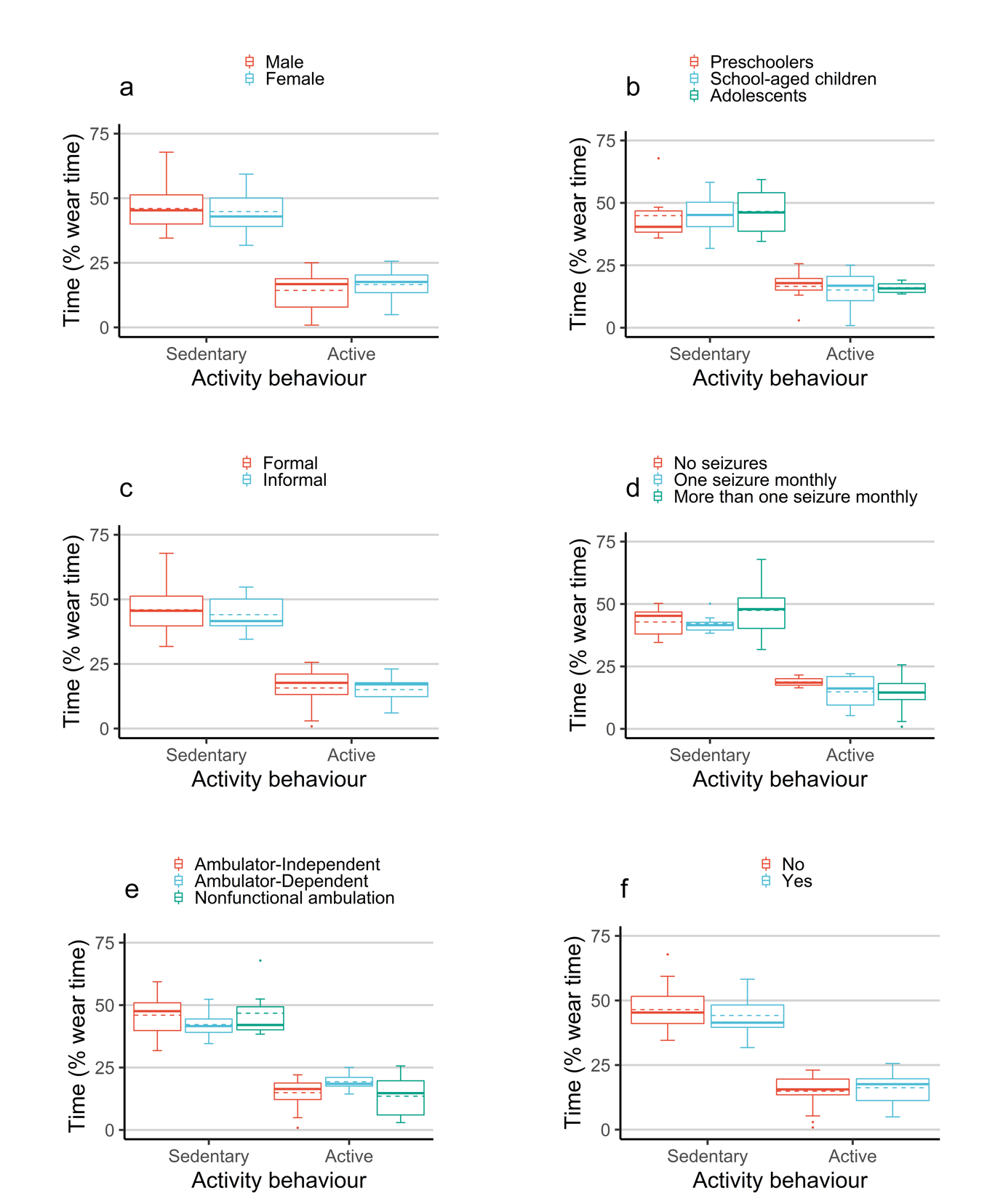


**Figure S3.** Box plots of wear time spent in sedentary behaviour or physical activity for participants according to (a) sex; (b) age group (preschoolers: 4-5 years, school-aged children: 6-13 years and adolescents: 14-16 years); (c) type of housing (formal and informal); (d) average seizures per month (none, one and more than one); (e) ambulation status (ambulator-independent, ambulator-dependent and nonfunctional ambulation); and (f) presence of mobility issues as reported in clinic. The boxes show, from bottom to top, the 25th percentile, median, and 75th percentile values, and the error bars indicate the lower and upper adjacent values. The upper adjacent value is defined as the largest data point ≤75th percentile + 1.5 interquartile range (IQR), and the lower adjacent value is defined as the smallest data point ≥25th percentile – 1.5 IQR. The outside values, which are data points more extreme than the upper and lower adjacent values, are individually plotted. The mean value is represented as a dashed line.
