# Supplementary material for: Personalized care of paediatric drug‐resistant epilepsy in Africa: A single‐centre pilot study utilizing mobile health and genetic testing
Source: Dev Med Child Neurol. 2025 Aug 20;68(3):394–406. doi: 10.1111/dmcn.16478 (PMC12875146; doi:10.1111/dmcn.16478)
Supplement: Supplementary file 4 — Figure S4: Box plots of time spent in nocturnal sleep or daytime nap for participants [file DMCN-68-394-s007.docx]

**Supplementary Figure S4**


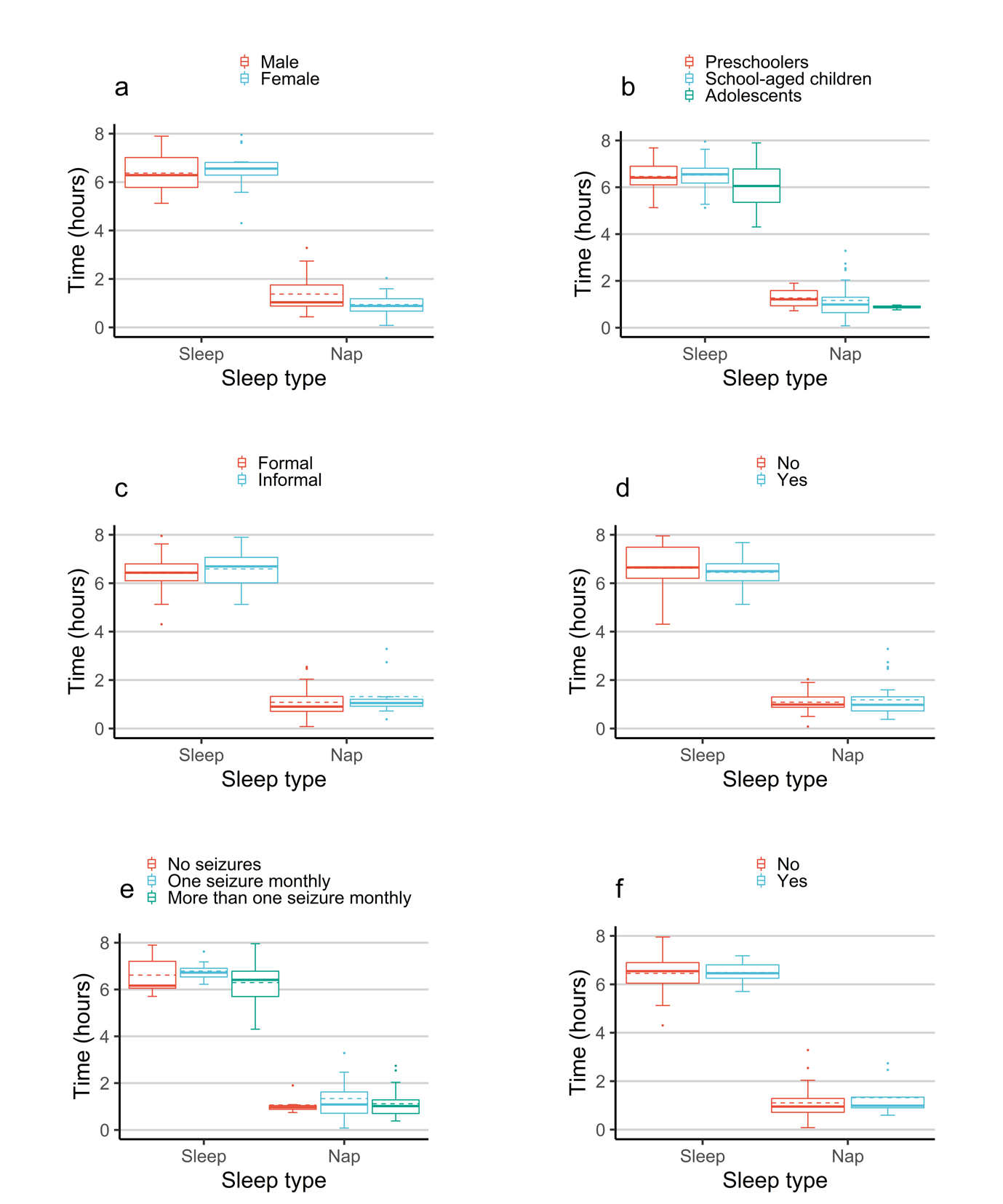


**Figure S4.** Box plots of time spent in nocturnal sleep or daytime nap for participants according to (a) sex; (b) age group (preschoolers: 4-5 years, school-aged children: 6-13 years and adolescents: 14-16 years); (c) type of housing (formal and informal); (d) bed sharing; (e) average seizures per month (none, one and more than one); and (f) presence of sleep issues as reported in clinic.
