# Supplementary material for: Personalized care of paediatric drug‐resistant epilepsy in Africa: A single‐centre pilot study utilizing mobile health and genetic testing
Source: Dev Med Child Neurol. 2025 Aug 20;68(3):394–406. doi: 10.1111/dmcn.16478 (PMC12875146; doi:10.1111/dmcn.16478)
Supplement: Supplementary file 6 — Table S1: mHealth technology‐based and clinical records data collected. [file DMCN-68-394-s003.docx]

**Supplementary Table S1:** mHealth technology-based and clinical records data collected

| **Data source/type** | **Data collected** |
| --- | --- |
| Wrist-worn watch data | - Number of steps and heart rate - Calculated sleep duration and type (light and deep sleep) from a 3-axis acceleration sensor |
| Customised app data | - Reporting of seizures either in real time or within a 48-hour window, including duration, type and management and supported video capture. - Illness or behavioural challenges captured using drop-down and free text fields - Daily yes/no pop-up question regarding the previous night’s sleep - Medication specific reminders when due, as entered by the medical practitioner - Caregiver recording of adherence and reasons for non-adherence. - Capturing of health-related visits - Monthly questionnaires on quality of life, function, behavioural challenges and sleep |
| Baseline demographic clinical data | - Age, sex, socio-economic and living conditions, ethnicity - Medical history, clinical diagnoses, comorbidities, family history, neurodevelopmental status - Drug-resistant epilepsy diagnosis, seizure history (frequency and types), - Past and current medications, medication adherence and access - Imaging findings, neurophysiological and metabolic investigations - Relative mobility of individuals via the Functional Ambulation Category (FAC)(Mehrholz et al., 2007; Holden et al 1984). |
| Clinic visit/follow-up file records | - Seizure frequency, duration and type - Sleep - Behaviour - Mobility (FAC score) - Learning or psychosocial concerns raised |
