# Supplementary material for: Personalized care of paediatric drug‐resistant epilepsy in Africa: A single‐centre pilot study utilizing mobile health and genetic testing
Source: Dev Med Child Neurol. 2025 Aug 20;68(3):394–406. doi: 10.1111/dmcn.16478 (PMC12875146; doi:10.1111/dmcn.16478)
Supplement: Supplementary file 7 — Table S2: Genes incorporated in gene panel design. [file DMCN-68-394-s005.docx]

**Supplementary Table S2:** Genes incorporated in gene panel design

| *ALDH7A1* | *GABRB1* | *MECP2* | *SLC35A2* |
| --- | --- | --- | --- |
| *ALG13* | *GABRB2* | *MEF2C* | *SLC6A1* |
| *ARX** | *GABRB3* | *NPRL2* | *SLC6A8* |
| *ATP1A2* | *GABRG2* | *NPRL3* | *SMC1A* |
| *ATP1A3* | *GNAO1* | *NR2F1** | *SNAP25* |
| *CACNA1A* | *GRIN1* | *PCDH19* | *STX1B* |
| *CACNA1E* | *GRIN2A* | *PNKP* | *STXBP1* |
| *CASK* | *GRIN2B* | *PNPO* | *SYN1* |
| *CDKL5* | *GRIN2D** | *POLG* | *SYNGAP1* |
| *CHD2* | *HCN1* | *PRRT2* | *SZT2* |
| *CLCN4* | *HNRNPU* | *PURA* | *TBC1D24* |
| *COL4A1* | *IQSEC2* | *SCN1A* | *TBL1XR1* |
| *DCX* | *KCNA2* | *SCN1B* | *TCF4* |
| *DEPDC5* | *KCNB1* | *SCN2A* | *TSC1* |
| *DNM1* | *KCNH5* | *SCN3A* | *TSC2* |
| *DYRK1A* | *KCNQ2* | *SCN8A* | *UBE3A* |
| *EEF1A2* | *KCNQ3* | *SIK1* | *WDR45* |
| *FOXG1* | *KCNT1* | *SLC13A5* | *WWOX* |
| *GABBR2* | *KIAA2022* | *SLC1A2* |  |
| *GABRA1* | *MBD5* | *SLC2A1* |  |
