# Supplementary material for: Personalized care of paediatric drug‐resistant epilepsy in Africa: A single‐centre pilot study utilizing mobile health and genetic testing
Source: Dev Med Child Neurol. 2025 Aug 20;68(3):394–406. doi: 10.1111/dmcn.16478 (PMC12875146; doi:10.1111/dmcn.16478)
Supplement: Supplementary file 9 — Table S4: Variants and respective genes used in custom mass array designed using the online database PharmGKB to identify variants and genes known to influence ASM metabolism in non‐African populations. [file DMCN-68-394-s002.docx]

**Supplementary Table S4:** Variants and respective genes used in custom mass array designed using the online database PharmGKB to identify variants and genes known to influence ASM metabolism in non-African populations

| **Variant** | **Gene** |
| --- | --- |
| rs2234922 | *EPHX1* |
| rs1800497 | *DRD2* |
| rs1799978 | *DRD2* |
| rs1414334 | *HTR2C* |
| rs2011425 | *UGT1A4* |
| rs1051740 | *EPHX1* |
| rs3812718 | *SCN1A* |
| rs489693 | *MC4R* |
