# Supplementary material for: Personalized care of paediatric drug‐resistant epilepsy in Africa: A single‐centre pilot study utilizing mobile health and genetic testing
Source: Dev Med Child Neurol. 2025 Aug 20;68(3):394–406. doi: 10.1111/dmcn.16478 (PMC12875146; doi:10.1111/dmcn.16478)
Supplement: Supplementary file 10 — Table S5: Proportion of levels of CHU9D by CHU9D dimension and by time of assessment. [file DMCN-68-394-s009.docx]

| **Supplementary Table S5**: Proportion of levels of CHU9D by CHU9D dimension and by time of assessment | | | | | | | | |
| --- | --- | --- | --- | --- | --- | --- | --- | --- |
| **CHU9D DIMENSION** | **CHU9D LEVEL*** | **Time of assessment** | | | | | | |
|  |  | **Baseline** | **Month 1** | **Month 2** | **Month 3** | **Month 4** | **Month 5** | **Month 6** |
|  |  | n (%) | n (%) | n (%) | n (%) | n (%) | n (%) | n (%) |
| **Worried** | Level 1  Level 2  Level 3  Level 4  Level 5  Not responded | 26 (66.7)  2 (5.1)  3 (7.7)  1 (2.6)  0 (0.0)  7 (17.9) | 17 (43.6)  4 (10.3)  1 (2.6)  1 (2.6)  0 (0.0)  16 (41.0) | 16 (41.0)  2 (5.1)  2 (5.1)  0 (0.0)  0 (0.0)  19 (48.7) | 15 (38.5)  2 (5.1)  0 (0.0)  0 (0.0)  1 (2.6)  21 (53.8) | 14 (35.9)  1 (2.6)  1 (2.6)  0 (0.0)  1 (2.6)  25 (56.4) | 14 (35.9)  2 (5.1)  1 (2.6)  0 (0.0)  0 (0.0)  22 (56.4) | 12 (30.8)  0 (0.0)  1 (2.6)  0 (0.0)  0 (0.0)  26 (66.7) |
| **Sad** | Level 1  Level 2  Level 3  Level 4  Level 5  Not responded | 28 (71.8)  1 (2.6)  2 (5.1)  1 (2.6)  1 (2.6)  6 (15.4) | 17 (43.6)  4 (10.3)  0 (0.0)  2 (5.1)  0 (0.0)  16 (41.0) | 15 (38.5)  2 (5.1)  2 (5.1)  0 (0.0)  1 (2.6)  19 (48.7) | 16 (41.0)  2 (5.1)  0 (0.0)  0 (0.0)  1 (2.6)  20 (51.3) | 14 (35.9)  2 (5.1)  0 (0.0)  1 (2.6)  0 (0.0)  22 (56.4) | 16 (41.0)  1 (2.6)  0 (0.0)  0 (0.0)  0 (0.0)  22 (56.4) | 12 (30.8)  0 (0.0)  1 (2.6)  1 (2.6)  0 (0.0)  25 (64.1) |
| **Pain** | Level 1  Level 2  Level 3  Level 4  Level 5  Not responded | 30 (76.9)  2 (5.1)  1 (2.6)  0 (0.0)  0 (0.0)  6 (15.4) | 19 (48.7)  3 (7.7)  2 (5.1)  0 (0.0)  0 (0.0)  15 (38.5) | 16 (41.0)  3 (7.7)  1 (2.6)  1 (2.6)  0 (0.0)  18 (46.2) | 17 (43.6)  1 (2.6)  1 (2.6)  0 (0.0)  1 (2.6)  19 (48.7) | 13 (33.3)  2 (5.1)  1 (2.6)  0 (0.0)  1 (2.6)  22 (56.4) | 16 (41.0)  1 (2.6)  0 (0.0)  0 (0.0)  0 (0.0)  22 (56.4) | 10 (25.6)  4 (10.3)  0 (0.0)  0 (0.0)  0 (0.0)  25 (64.1) |
| **Tired** | Level 1  Level 2  Level 3  Level 4  Level 5  Not responded | 20 (51.3)  6 (15.4)  3 (7.7)  2 (5.1)  2 (5.1)  6 (15.4) | 10 (25.6)  5 (12.8)  1 (2.6)  3 (7.7)  1 (2.6)  19 (48.7) | 16 (41.0)  3 (7.7)  0 (0.0)  1 (2.6)  1 (2.6)  18 (46.2) | 14 (35.9)  2 (5.1)  3 (7.7)  1 (2.6)  0 (0.0)  19 (48.7) | 11 (28.2)  3 (7.7)  2 (5.1)  2 (5.1)  0 (0.0)  21 (53.8) | 13 (33.3)  2 (5.1)  1 (2.6)  0 (0.0)  0 (0.0)  23 (59.0) | 7 (17.9)  5 (12.8)  2 (5.1)  0 (0.0)  0 (0.0)  25 (64.1) |
| **Annoyed** | Level 1  Level 2  Level 3  Level 4  Level 5  Not responded | 26 (66.7)  4 (10.3)  2 (5.1)  1 (2.6)  1 (2.6)  5 (12.8) | 13 (33.3)  6 (15.4)  3 (7.7)  1 (2.6)  1 (2.6)  15 (38.5) | 14 (35.9)  2 (5.1)  1 (2.6)  1 (2.6)  1 (2.6)  20 (51.3) | 16 (41.0)  3 (7.7)  0 (0.0)  1 (2.6)  0 (0.0)  19 (48.7) | 13 (33.3)  2 (5.1)  0 (0.0)  3 (7.7)  0 (0.0)  21 (53.8) | 13 (33.3)  3 (7.7)  1 (2.6)  0 (0.0)  0 (0.0)  22 (56.4) | 10 (25.6)  3 (7.7)  1 (2.6)  0 (0.0)  0 (0.0)  25 (64.1) |
| **Schoolwork/**  **homework** | Level 1  Level 2  Level 3  Level 4  Level 5  Not responded | 7 (17.9)  1 (2.6)  4 (10.3)  7 (17.9)  6 (15.4)  14 (35.9) | 5 (12.8)  1 (2.6)  4 (10.3)  2 (5.1)  5 (12.8)  22 (56.4) | 7 (17.9)  2 (5.1)  1 (2.6)  0 (0.0)  8 (20.5)  21 (53.8) | 7 (17.9)  3 (7.7)  2 (5.1)  1 (2.6)  7 (17.9)  19 (48.7) | 5 (12.8)  2 (5.1)  4 (10.3)  1 (2.6)  6 (15.4)  21 (53.8) | 7 (17.9)  2 (5.1)  2 (5.1)  2 (5.1)  4 (10.3)  22 (56.4) | 1 (2.6)  2 (5.1)  3 (7.7)  2 (5.1)  8 (20.5)  23 (59.0) |
| **Sleep** | Level 1  Level 2  Level 3  Level 4  Level 5  Not responded | 27 (69.2)  5 (12.8)  2 (5.1)  2 (5.1)  0 (0.0)  3 (7.7) | 18 (46.2)  2 (5.1)  3 (7.7)  0 (0.0)  1 (2.6)  15 (38.5) | 13 (33.3)  4 (10.3)  5 (12.8)  1 (2.6)  0 (0.0)  16 (41.0) | 17 (43.6)  4 (10.3)  0 (0.0)  0 (0.0)  0 (0.0)  18 (46.2) | 12 (30.8)  3 (7.7)  2 (5.1)  1 (2.6)  1 (2.6)  20 (51.3) | 15 (38.5)  2 (5.1)  0 (0.0)  0 (0.0)  0 (0.0)  22 (56.4) | 13 (33.3)  2 (5.1)  0 (0.0)  1 (2.6)  0 (0.0)  23 (59.0) |
| **Daily routine** | Level 1  Level 2  Level 3  Level 4  Level 5  Not responded | 19 (48.7)  4 (10.3)  1 (2.6)  2 (5.1)  6 (15.4)  7 (17.9) | 15 (38.5)  3 (7.7)  2 (5.1)  1 (2.6)  4 (10.3)  14 (35.9) | 13 (33.3)  1 (2.6)  1 (2.6)  1 (2.6)  4 (10.3)  19 (48.7) | 10 (25.6)  4 (10.3)  1 (2.6)  2 (5.1)  3 (7.7)  19 (48.7) | 12 (30.8)  4 (10.3)  1 (2.6)  1 (2.6)  1 (2.6)  20 (51.3) | 9 (23.1)  4 (10.3)  2 (5.1)  0 (0.0)  3 (7.7)  21 (53.8) | 8 (20.5)  2 (5.1)  5 (12.8)  0 (0.0)  0 (0.0)  24 (61.5) |
| **Able to join in activities** | Level 1  Level 2  Level 3  Level 4  Level 5  Not responded | 16 (41.0)  4 (10.3)  3 (7.7)  2 (5.1)  5 (12.8)  9 (23.1) | 12 (30.8)  4 (10.3)  4 (10.3)  3 (7.7)  2 (5.1)  14 (35.9) | 14 (35.9)  0 (0.0)  1 (2.6)  0 (0.0)  6 (15.4)  18 (46.2) | 8 (20.5)  4 (10.3)  2 (5.1)  2 (5.1)  3 (7.7)  20 (51.3) | 9 (23.1)  1 (2.6)  4 (10.3)  3 (7.7)  2 (5.1)  20 (51.3) | 9 (23.1)  1 (2.6)  6 (15.4)  1 (2.6)  1 (2.6)  21 (53.8) | 8 (20.5)  1 (2.6)  2 (5.1)  3 (7.7)  1 (2.6)  24 (61.5) |
| Level 1: I don’t feel/I don’t have any/I have no problems/ I can join in with any  Level 2: I feel a little bit/I have a little bit/I have a few problems/I can join in with most  Level 3: I feel a bit/I have a bit/I have some problems/I can join in with some  Level 4: I feel quite/I have quite a lot/I have many problems/I can join in with a few  Level 5: I feel very/I have a lot/I can’t do/I can join in with no | | | | | | | | |
