# Supplementary material for: Personalized care of paediatric drug‐resistant epilepsy in Africa: A single‐centre pilot study utilizing mobile health and genetic testing
Source: Dev Med Child Neurol. 2025 Aug 20;68(3):394–406. doi: 10.1111/dmcn.16478 (PMC12875146; doi:10.1111/dmcn.16478)
Supplement: Supplementary file 11 — Table S6: Proportion of levels 1, 2 and 3 by EQ‐5D‐Y dimension and by time of assessment and EQ VAS by time of assessment. [file DMCN-68-394-s008.docx]

**Supplementary Table S6**: Proportion of levels 1, 2 and 3 by EQ-5D-Y dimension and by time of assessment and EQ VAS by time of assessment

| **EQ-5D-Y DIMENSION** | **EQ-5D-Y LEVEL** | **Time of assessment** | | | | | | |
| --- | --- | --- | --- | --- | --- | --- | --- | --- |
|  |  | **Baseline** | **Month 1** | **Month 2** | **Month 3** | **Month 4** | **Month 5** | **Month 6** |
|  |  | n (%) | n (%) | n (%) | n (%) | n (%) | n (%) | n (%) |
| **Mobility** | No problems  Some problems  A lot of problems  Not responded | 17 (43.6)  14 (35.9)  6 (15.4)  2 (5.1) | 15 (38.5)  5 (12.8)  2 (5.1)  17 (43.6) | 11 (28.2)  3 (7.7)  4 (10.3)  21 (53.8) | 11 (28.2)  4 (10.3)  4 (10.3)  20 (51.3) | 9 (23.1)  4 (10.3)  4 (10.3)  22 (56.4) | 10 (25.6)  3 (7.7)  2 (5.1)  24 (61.5) | 7 (17.9)  5 (12.8)  3 (7.7)  24 (61.5) |
| **Looking after myself** | No problems  Some problems  A lot of problems  Not responded | 11 (28.2)  10 (25.6)  16 (41.0)  2 (5.1) | 5 (12.8)  7 (17.9)  9 (23.1)  18 (46.2) | 5 (12.8)  7 (17.9)  6 (15.4)  21 (53.8) | 6 (15.4)  6 (15.4)  7 (17.9)  20 (51.3) | 4 (10.3)  4 (10.3)  8 (20.5)  23 (59.0) | 2 (5.1)  8 (20.5)  4 (10.3)  25 (64.1) | 1 (2.6)  3 (7.7)  10 (25.6)  25 (64.1) |
| **Doing usual things** | No problems  Some problems  A lot of problems  Not responded | 14 (35.9)  12 (30.8)  8 (20.5)  5 (12.8) | 15 (38.5)  5 (12.8)  3 (7.7)  16 (41.0) | 12 (30.8)  4 (10.3)  2 (5.1)  21 (53.8) | 7 (17.9)  8 (20.5)  4 (10.3)  20 (51.3) | 9 (23.1)  7 (17.9)  1 (2.6)  22 (56.4) | 8 (20.5)  4 (10.3)  3 (7.7)  24 (61.5) | 4 (10.3)  6 (15.4)  4 (10.3)  25 (64.1) |
| **Having pain or discomfort** | No problems  Some problems  A lot of problems  Not responded | 24 (61.5)  11 (28.2)  1 (2.6)  3 (7.7) | 16 (41.0)  5 (12.8)  1 (2.6)  17 (43.6) | 14 (35.9)  5 (12.8)  1 (2.6)  19 (48.7) | 15 (38.5)  2 (5.1)  3 (7.7)  19 (48.7) | 14 (35.9)  2 (5.1)  0 (0.0)  23 (59.0) | 14 (35.9)  1 (2.6)  0 (0.0)  24 (61.5) | 8 (20.5)  6 (15.4)  0 (0.0)  25 (64.1) |
| **Feeling worried, sad or unhappy** | No problems  Some problems  A lot of problems  Not responded | 24 (61.5)  9 (23.1)  1 (2.6)  5 (12.8) | 17 (43.6)  4 (10.3)  1 (2.6)  17 (43.6) | 14 (35.9)  5 (12.8)  2 (5.1)  18 (46.2) | 13 (33.3)  3 (7.7)  2 (5.1)  21 (53.8) | 12 (30.8)  4 (10.3)  1 (2.6)  22 (56.4) | 11 (28.2)  3 (7.7)  0 (0.0)  25 (64.1) | 10 (25.6)  2 (5.1)  0 (0.0)  27 (69.2) |
| **EQ VAS (0-10)** | | **Baseline** | **Month 1** | **Month 2** | **Month 3** | **Month 4** | **Month 5** | **Month 6** |
| N | | 37 | 29 | 22 | 22 | 18 | 18 | 16 |
| Mean | | 8.1 | 7.5 | 7.6 | 7.1 | 7.7 | 8.3 | 7.4 |
| Standard deviation | | 2.8 | 2.4 | 2.9 | 3.2 | 2.2 | 2.2 | 2.9 |
| 25^th^ percentile | | 8.0 | 5.0 | 5.5 | 6.3 | 6.0 | 8.0 | 5.0 |
| 50^th^ percentile (median) | | 10.0 | 8.0 | 9.0 | 8.0 | 8.0 | 9.0 | 8.5 |
| 75^th^ percentile | | 10.0 | 9.0 | 10.0 | 9.8 | 9.8 | 10.0 | 10.0 |
