# Supplementary material for: Personalized care of paediatric drug‐resistant epilepsy in Africa: A single‐centre pilot study utilizing mobile health and genetic testing
Source: Dev Med Child Neurol. 2025 Aug 20;68(3):394–406. doi: 10.1111/dmcn.16478 (PMC12875146; doi:10.1111/dmcn.16478)
Supplement: Supplementary file 12 — Table S7: General pharmacogenomic (ADME) variant (n = 30) genotype counts, Hardy–Weinberg equilibrium P‐value, and Pearson's Chi‐squared test P‐value. [file DMCN-68-394-s001.docx]

**Supplementary Table S7:** General pharmacogenomic (ADME) variant (n=30) genotype counts, Hardy-Weinberg equilibrium P-value, and Pearson’s Chi-squared test P-value

| Gene | Variant | Genotype count | Genotype counts from African control population | HWE P-value | Pearson’s Chi-squared Test P-value |
| --- | --- | --- | --- | --- | --- |
| *ABCB1* | rs1045642 | n = 28  G/G = 22  A/G = 6  A/A = 0 | n = 661  G/G = 478  A/G = 168  A/A = 15 | 0.53 | 0.62 |
| *APOE* | rs429358 | n = 24  T/T = 15  C/T = 6  C/C = 3 | n = 661  T/T = 356  C/T = 256  C/C = 49 | 0.10 | 0.32 |
|  | rs7412 | n = 27  C/C = 20  C/T = 6  T/T = 1 | n = 661  C/C = 530  C/T = 126  T/T = 5 | 0.53 | 0.24 |
| *COMT* | rs4680 | n = 28  G/G = 10  G/A = 15  A/A = 3 | n = 661  G/G = 348  G/A = 255  A/A = 58 | 0.45 | 0.21 |
| *CYP1A2* | rs2069514 | n = 28  G/G = 18  G/A = 9  A/A = 1 | n = 661  G/G = 307  G/A = 294  A/A = 60 | 0.92 | 0.16 |
|  | rs762551 | n = 28  A/A = 11  C/A = 12  C/C = 5 | n = 661  A/A = 213  C/A = 317  C/C = 131 | 0.59 | 0.74 |
| *CYP2B6* | rs28399499 | n = 28  T/T = 24  C/T = 4  C/C = 0 | n = 661  T/T = 560  C/T = 93  C/C = 8 | 0.68 | 0.84 |
|  | rs3745274 | n = 28  G/G = 12  G/T = 13  T/T = 3 | n = 661  G/G = 259  G/T = 309  T/T = 93 | 0.85 | 0.86 |
| *CYP2C19* | rs4244285 | n = 28  G/G = 16  A/G = 12  A/A = 0 | n = 661  G/G = 459  A/G = 179  A/A = 23 | 0.15 | 0.14 |
|  | rs12248560 | n = 28  C/C = 19  C/T = 8  T/T = 1 | n = 632  C/C = 384  C/T = 243  T/T = 5 | 0.89 | 0.20 |
| *CYP2C9* | rs1057910 | n = 28  A/A = 26  C/A = 2  C/C = 0 | n = 661  A/A = 658  C/A = 3  C/C = 0 | 0.84 | N/A |
|  | rs7900194 | n = 28  G/G = 23  G/A = 5  A/A = 0 | n = 661  G/G = 591  G/A = 70  A/A = 0 | 0.60 | N/A |
|  | rs28371685 | n = 28  C/C = 27  C/T = 1  T/T = 0 | n = 661  C/C = 630  C/T = 30  T/T = 1 | 0.92 | 0.95 |
| *CYP2D6* | rs16947 | n = 28  G/G = 5  G/A = 11  A/A = 12 | n = 661  G/G = 143  G/A = 304  A/A = 214 | 0.39 | 0.51 |
|  | rs1135840 | n = 19  G/G = 8  C/G = 8  C/C = 3 | n = 661  G/G = 318  C/G = 258  C/C = 85 | 0.68 | 0.86 |
|  | rs3892097 | n = 27  C/C = 26  C/T = 1  T/T = 0 | n = 661  C/C = 589  C/T = 64  T/T = 8 | 0.92 | 0.48 |
|  | **r**s1065852 | n = 28  G/G = 24  A/G = 4  A/A = 0 | n = 661  G/G = 529  A/G = 115  A/A = 17 | 0.68 | 0.61 |
|  | rs28371706 | n = 12  G/G = 7  A/G = 5  A/A = 0 | n = 661  G/G = 420  A/G = 194  A/A = 47 | 0.36 | 0.47 |
|  | rs59421388 | n = 28  C/C = 19  T/C = 7  T/T = 2 | n = 661  C/C = 529  T/C = 122  T/T = 10 | 0.27 | **0.05** |
|  | rs28371725 | n = 28  C/C = 25  T/C = 3  T/T = 0 | n = 661  C/C = 639  T/C = 20  T/T = 2 | 0.76 | 0.08 |
| *CYP3A5* | rs776746 | n = 28  T/T = 23  T/C = 5  C/C = 0 | n = 661  T/T = 447  T/C = 190  C/C = 24 | 0.60 | 0.23 |
|  | rs41303343 | n = 28  D/D = 18  D/A = 10  A/A = 0 | n = 661  D/D = 516  D/A = 134  A/A = 11 | 0.25 | 0.12 |
|  | rs10264272 | n = 28  C/C = 20  C/T = 7  T/T = 1 | n = 661  C/C = 474  C/T = 170  T/T = 17 | 0.70 | 0.95 |
| *DRD2* | rs1800497 | n = 28  A/A = 5  A/G = 14  G/G = 9 | n = 661  A/A = 91  A/G = 327  G/G = 243 | 0.91 | 0.95 |
| *GLP1R* | rs1042044 | n = 28  A/A = 6  C/A = 11  C/C = 10  C/T = 1 | n = 661  A/A = 111  C/A = 322  C/C = 228  C/T = 0 | 0.38 | 0.66 |
|  | rs2300615 | n = 28  T/T = 22  G/T = 5  G/G = 1 | n = 661  T/T = 575  G/T = 83  G/G = 3 | 0.33 | 0.07 |
|  | rs6923761 | n = 28  C/C = 27  G/C = 1  G/G = 0 | n = 661  C/C = 643  G/C = 17  G/G = 1 | 0.92 | 0.93 |
| *MTHFR* | rs1801131 | n = 28  T/T = 21  G/T = 5  G/G = 2 | n = 661  T/T = 478  G/T = 166  G/G = 17 | 0.07 | 0.27 |
|  | rs1801133 | n = 28  G/G = 25  G/A = 3  A/A = 0 | n = 661  G/G = 549  G/A = 105  A/A = 7 | 0.76 | 0.64 |
| *VKORC1* | rs9923231 | n = 28  C/C = 24  C/T = 4  T/T = 0 | n = 661  C/C = 592  C/T = 66  T/T = 3 | 0.68 | 0.72 |
