# Supplementary material for: Personalized care of paediatric drug‐resistant epilepsy in Africa: A single‐centre pilot study utilizing mobile health and genetic testing
Source: Dev Med Child Neurol. 2025 Aug 20;68(3):394–406. doi: 10.1111/dmcn.16478 (PMC12875146; doi:10.1111/dmcn.16478)
Supplement: Supplementary file 13 — Table S8: ASM‐specific variant (n = 8) genotype counts, Hardy–Weinberg equilibrium (HWE) p‐value, and Pearson's Chi‐squared test p‐Value. [file DMCN-68-394-s004.docx]

**Supplementary Table S8: ASM-specific variant (n=8) genotype counts, Hardy-Weinberg equilibrium (HWE) p-value, and Pearson’s Chi-squared test p-Value**

| Gene | Variant | Genotype Count | Genotype Count in African Control Population | HWE P-value | Pearson’s Chi-squared Test P-value |
| --- | --- | --- | --- | --- | --- |
| *EPHX1* | rs1051740 | n = 28  T/T = 16  T/C = 9  C/C = 3 | n = 661  T/T = 492  T/C = 152  C/C = 17 | 0.34 | **0.02** |
| *HTR2C* | rs1414334 | n = 26  G/G = 15  G/C = 3  C/C = 8 | n = 661  G/G = 254  G/C = 176  C/C = 231 | **0.001** | 0.10 |
| *DRD2* | rs1799978 | n = 28  T/T = 16  T/C = 11  C/C = 1 | n = 661  T/T = 459  T/C = 175  C/C = 27 | 0.59 | 0.33 |
| *ANKK1* | rs1800497 | n = 28  G/G = 9  G/A = 14  A/A = 5 | n = 661  G/G = 243  G/A = 327  A/A = 91 | 0.91 | 0.79 |
| *UGT1A4* | rs2011425 | n = 28  T/T = 27  G/T = 1  G/G = 0 | n = 661  T/T = 562  G/T = 96  G/G = 3 | 0.92 | 0.24 |
| *EPHX1* | rs2234922 | n = 27  A/A = 17  G/A = 9  G/G = 1 | n = 661  A/A = 278  G/A = 299  G/G = 84 | 0.87 | 0.08 |
| *SCN1A* | rs3812718 | n = 24  C/C = 16  C/T = 5  T/T = 3 | n = 661  C/C = 287  C/T = 299  T/T = 75 | **0.04** | 0.05 |
| *NA – Intergenic variant* | rs489693 | n = 28  C/C = 8  C/A = 16  A/A = 4 | n = 661  C/C = 179  C/A = 298  A/A = 184 | 0.37 | 0.26 |
