# Supplementary material for: Personalized care of paediatric drug‐resistant epilepsy in Africa: A single‐centre pilot study utilizing mobile health and genetic testing
Source: Dev Med Child Neurol. 2025 Aug 20;68(3):394–406. doi: 10.1111/dmcn.16478 (PMC12875146; doi:10.1111/dmcn.16478)
Supplement: Supplementary file 14 — Appendix S1: Supplementary results [file DMCN-68-394-s010.docx]

**Appendix S1**

**Supplementary results**

*Self- or proxy-reported quality of life via mobile Patient Reported Outcomes (mPROs)*

Engagement with mPROs in this study has previously been reported (Davis et al 2021). Data regarding sleep and medication adherence most directly relevant to patient care is reported here.

*Sleep*

The caregivers reported a mean of 8 hours (SD 1.3) for sleep duration. Similar average sleep durations were reported for the children for whom sleep issues were previously reported in clinics and for the children without known sleep issues (p=0.79; Mann-Whitney test). The number of monthly seizures reported via the app did not impact on the reported average sleep duration (p=0.88; Kruskal-Wallis test).

On average, the caregivers reported that their child had poor sleep for 32.7% of the daily mPROs to which they responded. Poor sleep nights as reported by individuals ranged from 0% to 80.0% of the completed mPROs. According to the caregivers, the reasons why their child had trouble sleeping were mainly anxiety (34.4%), pain (18.7%) and restlessness (15.6%).

*Medication adherence*

All 39 participants’ caregivers completed at least one of the three requested medication adherence mPROs. In 38.5% of the answers, the caregivers indicated a good medication adherence. The reasons for medication non-adherence were running out of ASMs (14.7% of answers), wanting to stop ASMs (14.1%), not giving ASMs (11.9%), changing ASM dosages without consulting doctor (11.8%), giving ASMs less often as seizures had stopped (10.0%) and forgetting when and how to give ASMs (5.2%). Engagement with prompts for when ASM was due is reported in the previous paper.
